# Prokaryotic assemblages and metagenomes in pelagic zones of the South China Sea

**DOI:** 10.1186/s12864-015-1434-3

**Published:** 2015-03-20

**Authors:** Ching-Hung Tseng, Pei-Wen Chiang, Hung-Chun Lai, Fuh-Kwo Shiah, Ting-Chang Hsu, Yi-Lung Chen, Liang-Saw Wen, Chun-Mao Tseng, Wung-Yang Shieh, Isaam Saeed, Saman Halgamuge, Sen-Lin Tang

**Affiliations:** Bioinformatics Program, Taiwan International Graduate Program, Institute of Information Science, Academia Sinica, Taipei, Taiwan; Biodiversity Research Center, Academia Sinica, Taipei, Taiwan; Institute of Biomedical Informatics, National Yang-Ming University, Taipei, Taiwan; Institute of Oceanography, National Taiwan University, Taipei, Taiwan; Research Center for Environmental Changes, Academia Sinica, Taipei, Taiwan; Optimisation and Pattern Recognition Research Group, Department of Mechanical Engineering, Melbourne School of Engineering, The University of Melbourne, Victoria, Australia

**Keywords:** Metagenomics, Prokaryotic biodiversity, Ocean

## Abstract

**Background:**

Prokaryotic microbes, the most abundant organisms in the ocean, are remarkably diverse. Despite numerous studies of marine prokaryotes, the zonation of their communities in pelagic zones has been poorly delineated. By exploiting the persistent stratification of the South China Sea (SCS), we performed a 2-year, large spatial scale (10, 100, 1000, and 3000 m) survey, which included a pilot study in 2006 and comprehensive sampling in 2007, to investigate the biological zonation of bacteria and archaea using 16S rRNA tag and shotgun metagenome sequencing.

**Results:**

Alphaproteobacteria dominated the bacterial community in the surface SCS, where the abundance of Betaproteobacteria was seemingly associated with climatic activity. Gammaproteobacteria thrived in the deep SCS, where a noticeable amount of Cyanobacteria were also detected. Marine Groups II and III Euryarchaeota were predominant in the archaeal communities in the surface and deep SCS, respectively. Bacterial diversity was higher than archaeal diversity at all sampling depths in the SCS, and peaked at mid-depths, agreeing with the diversity pattern found in global water columns. Metagenomic analysis not only showed differential %GC values and genome sizes between the surface and deep SCS, but also demonstrated depth-dependent metabolic potentials, such as cobalamin biosynthesis at 10 m, osmoregulation at 100 m, signal transduction at 1000 m, and plasmid and phage replication at 3000 m. When compared with other oceans, urease at 10 m and both exonuclease and permease at 3000 m were more abundant in the SCS. Finally, enriched genes associated with nutrient assimilation in the sea surface and transposase in the deep-sea metagenomes exemplified the functional zonation in global oceans.

**Conclusions:**

Prokaryotic communities in the SCS stratified with depth, with maximal bacterial diversity at mid-depth, in accordance with global water columns. The SCS had functional zonation among depths and endemically enriched metabolic potentials at the study site, in contrast to other oceans.

**Electronic supplementary material:**

The online version of this article (doi:10.1186/s12864-015-1434-3) contains supplementary material, which is available to authorized users.

## Background

The application of high-throughput sequencing has accelerated the characterization of environmental prokaryotes, with two major approaches widely used for different purposes. One approach involves tag (multiplex) sequencing on phylogenetic marker genes (*e.g.*, 16S ribosomal RNA; rRNA) to understand community composition [1,2], whereas the other uses whole-genome shotgun sequencing on environmental DNA (*i.e.*, metagenome) to study metabolic potentials embedded in the environment [3-5].

With the use of 16S rRNA tag sequencing, the immense diversity of marine prokaryotic communities has been reported in various oceanic habitats, including sea surfaces [6,7], bathypelagic zones [1,8], and deep-sea hydrothermal vents [2]. Several environmental parameters have been associated with marine prokaryotic diversity, such as substrate availability [9], day length [10], and water mass [11]. Prokaryotic communities also exhibited compositional variation at various pelagic depths [12], confirming the importance of depth in determining their distribution in the ocean.

A metagenomic study using whole-genome shotgun sequencing describes marine prokaryotic diversity from a metabolic perspective. Analyses on metagenomes collected in the water column of the Hawaii Ocean Time-series (HOT) station ALOHA revealed vertical zonation of protein functions [5], whereas the deep-sea community had greater metabolic versatility and genomic plasticity than sea-surface counterparts [13]. Based on genomes of 137 prokaryotic isolates from global ocean surfaces, there was a preference for slow growth in most cosmopolitan lineages of dominant abundance, whereas minor species apparently switched between slow and fast growth under ambient conditions [14]. Functional adaptation of marine prokaryotes was manifested by the enrichment of specific metabolic pathways in various environments, such as genes for microbial heterotrophy in the deep Mediterranean Sea [15], transposases in a hydrothermal chimney biofilm [16], and transporters in a hadopelagic metagenome [17].

The South China Sea (SCS), the largest marginal sea in the tropics, is a preferred area for oceanographic research, owing to its complicated basin topography and current system [18,19]. The diversity of several prokaryotic groups in the SCS has been reported. For example, flow cytometric analysis on the SCS surface demonstrated the dominance of *Prochlorococcus* in summer, whereas *Synechococcus* and picoeukaryotes had peak abundance in winter [20]. Based on *nifH* gene sequences, the diversity of the diazotroph community in the SCS was relatively simple, predominated by *Trichodesmium* and Alphaproteobacteria [21]. Compositional differences and functional gene diversity of the SCS Crenarchaeota community indicated niche partitioning in the water column [22]. Based on 16S rRNA tag sequencing, bacterial communities in the SCS contained abundant SAR11 bacteria at depths and had depth-dependent compositions [23]. The SCS is a marginal sea connected to the Pacific Ocean at the surface, and has a deep basin with persistently strong stratification [18], both of which make this oceanic area an isolated niche under mesopelagic depths. Similar to the observed depth-dependent community composition, we further hypothesized that the metabolic potentials of local prokaryotes are also zoned within the pelagic SCS. At the same time, we were also intrigued by many questions about metabolic potentials of local prokaryotic communities. What metabolic functions characterize different pelagic zones in the SCS? What metabolic potentials in the SCS are different from and similar to other oceans at different depths?

To verify this hypothesis and address these questions, a 2-year metagenomics survey was conducted at the South East Asia Time-series Study (SEATS) station (18°15′N, 115°30′E). In October 2006, a pilot study was performed to identify the pelagic depths harboring differential bacterial communities. In October 2007, detailed sampling was conducted at those depths to probe the bacterial and archaeal communities (using 16S rRNA tag sequencing) and metabolic potentials (using shotgun metagenome sequencing). Thereafter, comparisons among the SCS and other oceans were conducted to identify contrasting features delineating pelagic zones around the globe.

## Results

To determine appropriate sampling depths for our metagenomics survey, a pilot study was conducted at the SEATS station on October 20–21, 2006, with detailed experimental procedures appended in the Supplementary Methods (Additional file 1). In brief, seawater samples were intensively collected at 15 depths throughout the water column (10–2000 m). Denaturing gradient gel electrophoresis of bacterial 16S rRNA genes identified similar community patterns at epipelagic depths (10–80 m), whereas there was a distinct community at 100 m (Additional file 1: Figure S1). Therefore, we selected 10 m (epipelagic zone), 100 and 1000 m (mesopelagic layer), and 3000 m (bathypelagic layer) for the 2007 metagenomics survey.

### Hydrography and microbial abundance in the South China Sea

Major sampling was conducted during Cruise 845 of the R/V *Ocean Research I* of the Oceanography Institute of National Taiwan University on October 21–30, 2007. Four seawater samples (at 10, 100, 1000, and 3000 m) were collected from the SEATS station, and stratified profiles of water temperature, salinity, and density were measured simultaneously with a Sea-Bird conductivity-temperature-depth profiler (Additional file 1: Figure S2). The temperature-salinity diagram indicated that there were three water masses in the sampled water column (Additional file 1: Figure S3). Several nutrient parameters were also measured [24,25] (Additional file 1: Table S1). Detailed comparisons of SCS hydrography with other oceans are reported in the Supplementary Results (Additional file 1). Microbial abundance in the SCS was highest at 100 m and lowest at 3000 m (inferred by enumerating microbial particles of 0.22–10 μm in diameter using SYBR Gold staining, Additional file 1: Table S1).

### Prokaryotic 16S rRNA gene diversity in the South China Sea

In this study, tag sequencing [26] of the 16S rRNA hypervariable V6 region (abbreviated as V6) was used to characterize bacterial and archaeal community diversity and composition. After length-filtration and primer removal, four bacterial and four archaeal amplicon samples were submitted to the SILVA-ngs pipeline [27] to define the operational taxonomic units (OTUs) at 98% similarity level with taxonomic labels from SILVA. Diversity indices and rarefaction curves were estimated per sample using Mothur [28] based on OTUs generated by SILVA-ngs.

Among four sampling depths, bacterial and archaeal communities both had their greatest diversity (based on the Shannon index) at 100 m and lowest at 3000 m (Table 1). According to rarefaction curves, only the bacterial sample at 3000 m approached an asymptote (Additional file 1: Figure S4), suggesting that additional sequencing efforts in the shallower zones would detect greater diversity. In the SCS, the bacterial community was always more diverse than Archaea within the same pelagic zone, consistent with previous studies that used a clone library [29] or 16S rRNA tag sequencing [2]. Good’s coverage estimates sampling completeness by calculating the probability that a randomly selected read from a sample had been sequenced. At 98% similarity level, Good’s coverage values for bacterial and archaeal samples ranged from 0.94 to 0.976 when estimated using all reads (Additional file 1: Table S2).

**Table 1:** **Bacterial and archaeal diversity indices based on 16S rRNA gene libraries of the SEATS station**

**Samples** ^***a***^	**N**	**# OTU** ^***b***^	**# Singleton OTU**	**Shannon**	**Simpson**	**Chao 1**	**Evenness** ^***c***^	**Richness** ^***d***^	**Good’s coverage** ^***e***^
Bac 10 m	675^*f*^	218	130	4.561	0.025	467	0.85	45.59	0.807
Bac 100 m	675	240	150	4.699	0.021	544	0.86	52.66	0.778
Bac 1000 m	675	197	127	4.307	0.03	484	0.82	44.53	0.812
Bac 3000 m	675	134	67	3.997	0.033	246	0.82	23.33	0.901
Arc 10 m	675	87	33	3.606	0.044	136	0.81	11.31	0.951
Arc 100 m	675	101	42	3.733	0.041	157	0.81	14.49	0.938
Arc 1000 m	675	96	39	3.535	0.06	153	0.77	13.43	0.942
Arc 3000 m	675	82	29	3.335	0.063	108	0.76	9.9	0.957

^*a*^Bac: Bacteria, Arc: Archaea.

^*b*^OTUs are defined at the 98% sequence similarity using 16S rRNA hypervariable V6 region.

^*c*^Evenness is defined as Shannon/ln(# OTU).

^*d*^Richness is defined as (# singleton OTU-1)/log_10_N. The maximum value is (N-1)/log_10_N.

^*e*^Good’s coverage is defined as 1-(# singleton OTU)/N.

^*f*^The read number is rarefied to the minimum sample size of all compared samples by resampling with 1000 iterations. Data derived from all reads per sample are available in Additional file 1: Table S2.

### Bacterial community structure in the South China Sea

Bacterial and archaeal community structures were inferred from the taxonomy information of all OTUs identified by the SILVA-ngs pipeline via the BLASTn search of OTU representatives against the SILVA SSU Ref database.

**Figure 1:**
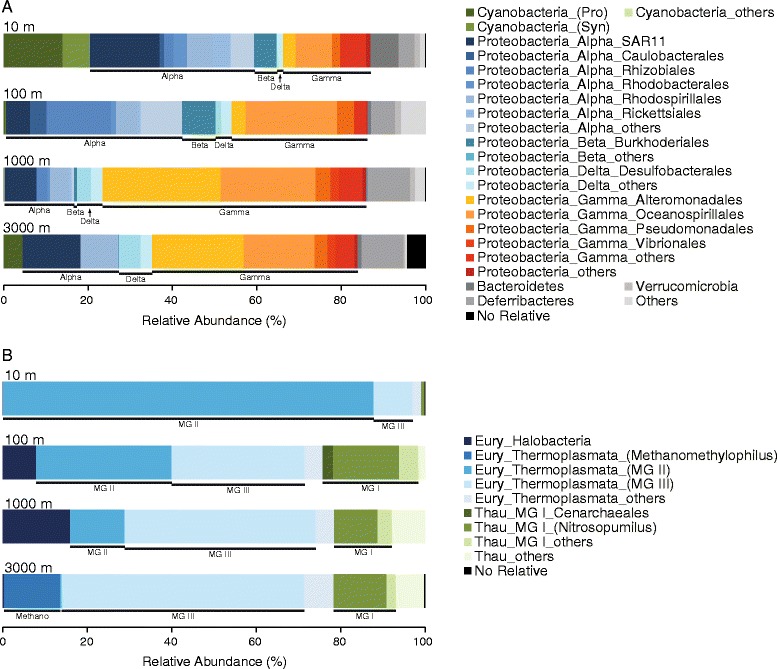
**Taxonomic composition of (A) bacterial and (B) archaeal communities identified in 16S rRNA gene-tagged sequences.** Bacterial taxa with >1% relative abundance on average are displayed and named in the format phylum_class_order. Taxon names deeper than order level are listed in parenthesis. Abbreviations: Alpha, Alphaproteobacteria; Beta, Betaproteobacteria; Delta, Deltaproteobacteria; Eury, Euryarchaeota; Gamma, Gammaproteobacteria; MG, Marine Group; Pro, *Prochlorococcus*; Syn, *Synechococcus*; Thau, Thaumarchaeota.

With regard to the bacterial community, the Proteobacteria were predominant in the SCS water column, accounting for 66.5, 85.6, 85.7%, and 79.4% of total 16S rRNA V6 amplicon sequences in the 10-, 100-, 1000-, and 3000-m samples, respectively. Cyanobacteria were relatively abundant (20.5%) at 10 m. At the class level, Alpha- and Gammaproteobacteria were the two most abundant lineages, albeit with opposite distributions with depth (Figure 1A). Betaproteobacteria (mostly Burkholderiales) contributed 5.4 and 7.9% of the 10- and 100-m bacterial communities, respectively, whereas Gamma- and Deltaproteobacteria became more abundant at ≥1000 m (Figure 1A). Cyanobacteria not only accounted for a substantial abundance at 10 m, but interestingly accounted for 4.5% of the 3000-m bacterial community (Figure 1A).

### Archaeal community structure in the South China Sea

Euryarchaeota and Thaumarchaeota were the two major phyla of the archaeal community in the SCS (Figure 1B). Within the Euryarchaeota phylum, Marine Groups (MG) II and III were inversely distributed through the water column. MG II Euryarchaeota gradually decreased in abundance (from 87.7 to 0.4%) at depths from 10 to 3000 m, whereas MG III Euryarchaeota increased from 9.2 to 57.4% (Figure 1B). In addition, MG I Thaumarchaeota, which were previously affiliated with the Crenarchaeota phylum [30], were mostly comprised of Nitrosopumilales. They were most abundant at depths of ≥100 m, with relatively stable abundances (13.6–22.6%) in comparison to the Euryarchaeota (Figure 1B). The decreasing abundance of MG II Euryarchaeota with increasing depth and detection of MG I Thaumarchaeota below 100 m correspond to previous observations using CARD-FISH [31].

### Depth specificity of prokaryotes in the South China Sea

**Figure 2:**
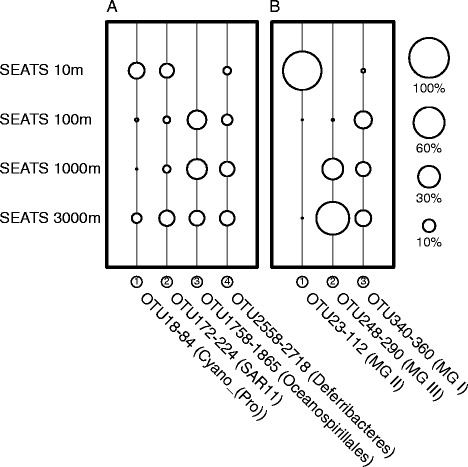
**Depth-specific OTU groups of (A) bacterial and (B) archaeal communities.** The serial number of OTU groups (in circle), OTU numbers, and taxonomy (in parenthesis) all correspond to the labels in Figure S5 and S6 in Additional file 1. The area of each bubble represents the cumulative relative abundance in the sample examined. Abbreviations: Cyano, Cyanobacteria; MG, Marine Group; Pro, *Prochlorococcus*.

Depth specificity of OTUs occurred at various pelagic zones in the SCS. A subset of cyanobacterial OTUs appeared at 10 and 3000 m, whereas a group of OTUs affiliated with Oceanospirillales only existed below 100 m depth (Figure 2A). By aligning OTUs according to taxonomy across all depths, the Oceanospirillales order consisted of different OTUs according to depth (Additional file 1: Figure S5), and eurybathic taxa such as SAR11 and Deferribacteres also comprised different depth-specific OTUs (Figure 2A; Additional file 1: Figure S5). Furthermore, MG II and MG III Euryarchaeota had more prominent depth specificity at shallow and deep depths, respectively (Figure 2B; Additional file 1: Figure S6). Although MG I Thaumarchaeota harbored similar abundances at all depths below 100 m (Figure 2B), there was also depth-specific variation in OTUs within the phylum (Additional file 1: Figure S6).

### Comparison of 16S rRNA gene diversity with other oceans

Eleven bacterial 16S rRNA V6 tag sequencing libraries were collected from three water columns at the Azores (37°20′N, 18°53′W), Mediterranean Sea (40°39′N, 2°51′E), and HOT (22°45′N, 158°0′W) to compare 16S rRNA gene diversity with the SCS. The three water columns were chosen because they contained samples covering epipelagic, mesopelagic, and bathypelagic depths. All samples were analyzed using the SILVA-ngs pipeline and Mothur, to match the analysis of all SCS datasets.

**Figure 3:**
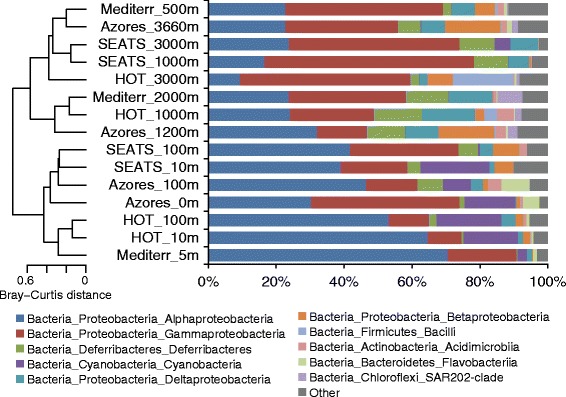
**Clustering analysis of bacterial communities in the SCS and other oceans.** Hierarchical clustering was performed using complete linkage on Bray–Curtis distance measures of 94 bacterial classes assigned to tag sequences of 16S rRNA hypervariable V6 region. Only the abundant classes (averaged from all samples) are included in the stacked bar chart for clarity. The remaining classes are collapsed into one group denoted as “Other”.

In the four water columns, bacterial community diversity (as represented by the Shannon index) always peaked at intermediate depths, with the lowest value at the surface (Table 2; Additional file 1: Table S3). Regarding taxonomic composition, Alphaproteobacteria and Cyanobacteria were more abundant in all of the ocean surfaces examined, whereas Gammaproteobacteria were more common in the deeper oceans. These results corresponded to the clustering results showing that bacterial communities in shallow waters separated from their deep-water counterparts (Figure 3). Similar clustering patterns were apparent in the non-metric multidimensional scaling analysis of bacterial communities at both the class and genus levels (Additional file 1: Figure S7).

**Table 2:** **Bacterial diversity indices based on 16S rRNA gene libraries of the SEATS station and other oceans**

**Samples**	**N**	**# OTU** ^***a***^	**# Singleton OTU**	**Shannon**	**Simpson**	**Chao 1**	**Evenness** ^***b***^	**Richness** ^***c***^	**Good’s coverage** ^***d***^
Azores 0 m	6711^*e*^	616	322	4.412	0.039	1213	0.69	83.88	0.952
Azores 100 m	6711	863	455	4.821	0.041	1676	0.71	118.64	0.932
Azores 1200 m	6711	1170	574	5.373	0.024	1908	0.76	149.73	0.914
Azores 3660 m	6711	1099	621	5.01	0.036	2213	0.72	162.02	0.907
HOT 10 m	6711	508	221	3.858	0.084	787	0.62	57.49	0.967
HOT 100 m	6711	710	347	4.561	0.043	1278	0.69	90.42	0.948
HOT 1000 m	6711	1106	624	5.294	0.018	2152	0.76	162.8	0.907
HOT 3000 m	6711	590	274	4.298	0.043	964	0.67	71.34	0.959
Mediterr 5 m	6711	321	129	3.882	0.051	507	0.67	33.45	0.981
Mediterr 500 m	6711	820	320	5.039	0.026	1183	0.75	83.36	0.952
Mediterr 2000 m	6711	1012	544	5.091	0.029	1996	0.74	141.89	0.919
SEATS 10 m	6711	748	367	4.936	0.025	1309	0.75	93.03	0.947
SEATS 100 m	6711	841	402	5.124	0.021	1491	0.76	104.79	0.94
SEATS 1000 m	6711	733	356	4.668	0.03	1253	0.71	92.77	0.947
SEATS 3000 m	6711	413	198	4.203	0.033	730	0.7	51.48	0.971

^*a*^OTUs are defined at the 98% sequence similarity using 16S rRNA hypervariable V6 region.

^*b*^Evenness is defined as Shannon/ln(# OTU).

^*c*^Richness is defined as (# singleton OTU-1)/log_10_N. The maximum value is (N-1)/log_10_N.

^*d*^Good’s coverage is defined as 1-(# singleton OTU)/N.

^*e*^The read number is rarefied to the minimum sample size of all compared samples by resampling with 1000 iterations. Data derived from all reads per sample are available in Additional file 1: Table S3.

### Metagenomics of the South China Sea

Metagenomes from four depths in the SCS were sequenced using a whole-genome shotgun approach. A total of 970,172 metagenomic reads were generated using Roche 454 pyrosequencing and assembled into 85,277 contigs by GS *de novo* assembler (40-bp minimum overlap at 99% consensus). A total of 78,602 open reading frames (ORFs) were predicted from contigs, of which the average coverage ranged from 3.02x to 5.25x (Table 3). Approximately 61% of the ORFs matched protein homologs in the eggNOG database, using BLASTp with an *e*-value ≤10^−5^. On average, 27% of the ORFs passed the second criterion of a bit-score ≥100 (Table 3).

**Table 3:** **Compositional and statistical summary of SEATS metagenomes**

**Characteristics**	**10 m**	**100 m**	**1000 m**	**3000 m**
Total reads	228154	270377	248730	222911
Total read length (bp)	52534435	63904461	58780946	51977555
Average read length (bp)	230	236	236	233
Total contigs	15914	24881	21425	23057
Total contig length (bp)	3590518	5694438	6496827	5425432
Total reads on contig	47099	77074	144516	81612
Average coverage per contig	3.02x	3.19x	5.25x	3.50x
% G + C of contigs	37.6	39.7	46.3	47.0
Total ORFs	14346	22888	21362	20006
% eggNOG hits (*e* ≤10^−5^)	64.5	58.9	60.1	62.0
% eggNOG hits (*e* ≤10^−5^, bits ≥100)	29.4	22.1	30.1	26.4
Average genome size ± sd (Mbp)	1.72 ± 0.01	1.84 ± 0.03	3.95 ± 0.03	3.42 ± 0.03

**Figure 4:**
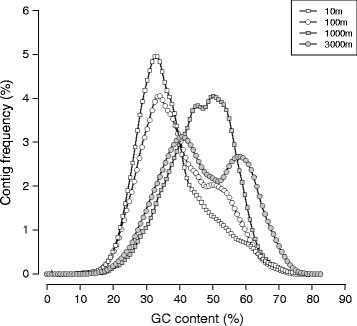
**GC content distribution of SEATS metagenomes.** Curves are kernel density estimates of the %GC values of contigs in the metagenome.

The metagenomic GC content (%GC) at different depths was variable, reflecting distinct genomic compositions at each of the four depths. Metagenomes in the shallow SCS (10 and 100 m) had 10% lower %GC values than at depth (1000 and 3000 m; Table 3). The 3000-m metagenome had two peaks (at 40 and 60%) in the %GC plot (Figure 4), indicating co-existence of low- and high-GC microbial groups. The 10- and 100-m metagenomes were estimated to have average genome sizes of 1.72 and 1.84 Mbp, respectively, roughly two-fold smaller than the other two deep SCS samples (Table 3).

Based on reciprocal tBLASTx analysis, each metagenome had, on average, 22.6% of contigs that matched other samples. Homologous contigs were more frequently detected at neighboring depths; in this regard, 24.6% of contigs at 3000 m matched the 1000-m sample, whereas only 14.7% matched the 10-m contigs (Additional file 1: Table S4).

### Functional analyses of metagenomes in the South China Sea

Putative ORFs were searched against the eggNOG database to annotate their function, based on the Clusters of Orthologous Groups (COGs) system. Only COG assignments with enough significance (*e*-value ≤10^−5^) and alignment quality (bit-score ≥100) were involved in the functional enrichment analysis using the R package ShotgunFunctionalizeR [32]. The COG gene family frequency was quantified using the number of reads mapped to ORFs. Every metagenome was compared with all the others (direct comparison of two groups; one versus the other three) to identify significantly enriched COGs (Benjamini-Hochberg adjusted *P*-value ≤0.05) at each depth.

**Figure 5:**
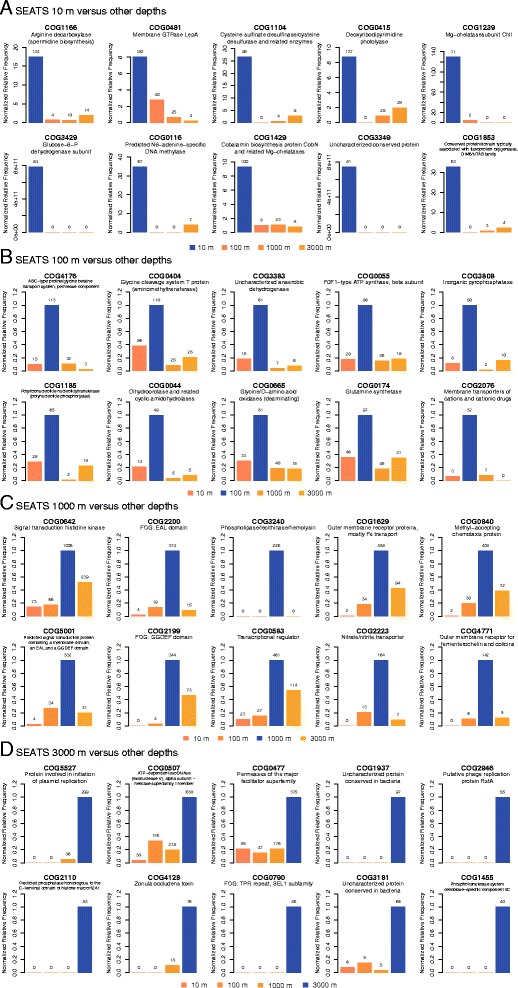
**Enriched functional genes at (A) 10-, (B) 100-, (C) 1000-, and (D) 3000-m pelagic zones at the SEATS station.** The original counts of each functional gene are labeled on top of each bar.

In the 10-m metagenome, genes for bacteriochlorophyll synthesis (COG1239), antioxidant regulation (COG3429), photolyase (COG0415), DNA synthesis (COG1429), and amino acid metabolism (COG1166 and COG1104) were enriched (Figure 5A). Osmoregulation functions were relatively abundant at 100 m, especially for osmolyte transportation (COG4176) and catabolism (COG0404 and COG0665; Figure 5B). Other enriched functions included COGs for nitrogen assimilation (COG0174) and energy production (COG3808 and COG0055). The 1000-m metagenome featured abundant COGs for signal transduction (COG5001, COG0642, COG2200, COG2199), chemotaxis (COG0840), and iron acquisition (COG1629 and COG4771), whereas plasmid and phage replication initiation (COG5527 and COG2946), nutrient transport (COG0477 and COG1455), and uncharacterized conserved bacterial proteins (COG3181 and COG1937) were enriched in the 3000-m metagenome (Figure 5D).

As oceanographic parameters had similar vertical profiles with depth (Additional file 1: Figure S8) and were highly correlated (*r*^2^ = 0.86 ± 0.11; Additional file 1: Table S5), depth was selected as the parameter for Poisson regression analysis of COG abundances. Overall, COGs enriched in the surface SCS had decreasing abundance with increasing depth (Additional file 1: Figure S9), and the opposite trend was found in COGs that were abundantly detected at 1000 and 3000 m (Additional file 1: Figure S10). Details are appended in the Supplementary Results (Additional file 1).

### Comparison of functional genes in other oceanic metagenomes

A comparison between SCS metagenomes and metagenomes from other oceans was performed to examine metabolic genes regionally enriched in the surface (10-m) and deep (3000-m) SCS. We also applied the two-group comparison (*i.e.*, the SCS versus other oceans) for this analysis. Gene family frequency in each metagenome was quantified using the number of reads mapped to ORFs, which were functionally annotated by BLASTp using the same criteria as the SCS metagenomes (*e*-value ≤10^−5^, bits-score ≥100, the eggNOG database). Significantly enriched COG gene families were identified by the Benjamini-Hochberg adjusted *P*-value ≤0.05 [32].

**Figure 6:**
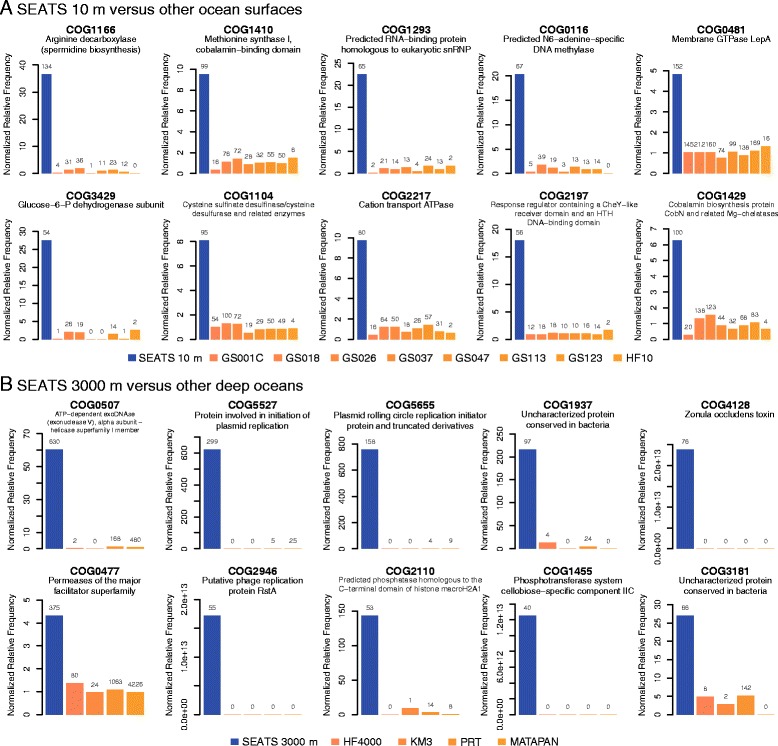
**Enriched functional genes at (A) SEATS 10 m versus other ocean surfaces and (B) SEATS 3000 m versus other deep oceans.** The original counts of each functional gene are labeled at the top of each bar.

In addition to the cobalamin synthesis protein (COG1429) and arginine decarboxylase (COG1166), the SCS surface also contained more methionine synthase (COG1410), glucose-6-P dehydrogenase (COG3429), and membrane GTPase (COG0481) than other ocean surfaces (Figure 6A). Interestingly, urease (COG0804, ranked 18th) was also more abundant at the SCS surface. For the deep SCS, there were substantial COGs of exonuclease V (COG0507), plasmid replication initiation (COG5527 and COG5655), phage replication protein (COG2946), and nutrient transport (COG0477 and COG1455), all of which had informative annotations in the top-10 list (Figure 6B).

Last, global sea-surface and deep-sea metagenomes (including SCS and other oceans) were compared to identify metabolic genes discerning the epi- and bathypelagic zones. The COGs for ammonium assimilation (COG0404, COG0069, and COG0665), carbon assimilation (COG4664, COG4663, and COG0451), and carotene production (COG1233) prevailed in the global ocean surfaces (Additional file 1: Figure S11 and Table S6). In global deep-sea metagenomes, transcriptional regulators (COG0583, COG1309, and COG1609), signal transduction proteins (COG0642, COG2199, and COG2200), and transposases (COG2801, COG3436, COG3547, COG4584, and COG4644) were detected in abundance (Additional file 1: Figure S12 and Table S7).

## Discussion

The 16S rRNA tag sequencing [1,2] and shotgun metagenome analysis [3-5] are two important methods that have greatly enhanced microbial community surveys. In this study, we used both approaches to profile prokaryotic communities and metagenomes in the SCS water column at depths of 10, 100, 1000, and 3000 m. Comparative analyses of the SCS and other oceans described zonation of microbial community and metabolic potentials in global epi- and bathypelagic zones.

Although Betaproteobacteria is a typical freshwater lineage [33], it is frequently present in oceans [7,11,12,34]. Betaproteobacteria in the SCS mainly appeared at 10 and 100 m, where it accounted for 5.4 and 7.9% of local communities, respectively (Figure 1A). Phylogenetic analysis revealed that they were closely related to the genera *Cupriavidus, Ralstonia*, and *Herbaspirillum* (Additional file 1: Figure S13). Betaproteobacteria in the SCS was more abundant than the reported 3.5% in the Arctic Ocean [7]. The abundance of Betaproteobacteria was attributed to the geographical location of our sampling site. The SEATS station is adjacent to the Pearl River, the second largest river by volume in China, and offshore freshwater inflow could be one of the sources of Betaproteobacteria in the SCS. Furthermore, the SCS has frequent typhoon events during the summer and autumn, which increases the Betaproteobacteria abundance in coastal waters [35]; therefore, the abundant Betaproteobacteria in the SCS surface could be a regional community feature associated with climatic activity. Jing et al. [23] reported a minor amount of Betaproteobacteria (<1%) in the SCS surface during August 2007, which was lower than our observation in October 2007 at the same site. Two potential reasons could contribute to this variation. Firstly, Jing et al. used a different region to probe the community (bacterial 16S V3–V4) [23], and secondly, a typhoon (Pabuk, August 5–9, 2007) and a tropical storm (Francisco, September 23–25, 2007) crossed the northern SCS between Jing et al’s sampling and the present study.

Following the scenario that coastal inflow might introduce bacteria into the open SCS, the low abundance of Flavobacteriia (a bacterial lineage reportedly abundant in coastal waters [36,37]) drew our attention. The primer coverage analysis revealed that the known diversity of Bacteroidetes (phylum) and Flavobacteriia (class) were barely covered by the primer we used in this study (Additional file 1: Table S8), suggesting a potential underestimation of the diversity of Bacteroidetes (including Flavobacteriia) at the study site.

Cyanobacteria harvest light energy through photosynthesis and thrive at the ocean surface. Therefore, detection of Cyanobacteria in the SCS 3000 m (approximately 4.5%) was unexpected (Figure 1A), although it is not a new finding for aphotic areas such as deep-sea hydrothermal vents [2], at 770 m in the North Pacific Subtropical Gyre [38], and at 800 and 4400 m in the North Pacific Ocean [12]. A bacterial community survey detected Cyanobacteria at 2000 m in the SCS [23], and another report indicated that there was *Prochlorococcus* in the aphotic western Pacific Ocean [39]. They ascribed the presence of *Prochlorococcus* in aphotic waters to physical processes that cause vertical water mixing. However, the higher abundance of Cyanobacteria at 3000 m relative to intermediate depths (100 and 1000 m) suggested their potential presence as a long-term lineage in SCS bottom waters. If so, questions about deep-sea Cyanobacteria (*e.g.* their metabolic activity and trophic strategy) warrant further investigation. The heterotrophic ability of *Prochlorococcus* has been reported, such as the uptake and use of amino acids [40], dimethylsulfoniopropionate [41], phosphite [42], and glucose [43]. However, the low availability of dissolved organic matter in the deep sea makes it more likely for *Prochlorococcus* to use endogenous carbon sources (*e.g.*, carbohydrates and lipids) for survival in the dark, just like other cyanobacteria [44]. Moreover, these phototrophs are also likely dormant or even dead in the deep sea.

It was noteworthy that Euryarchaeota dominated the archaeal community in the SCS water column, with more MG II Euryarchaeota at 10 m (87.7%) and MG III Euryarchaeota in the 3000 m water (57.4%). MG II Euryarchaeota were generally abundant at the ocean surface [5,45]; their decreasing abundance with depth has been reported at the HOT station ALOHA [46], SEATS station [31], and Arctic Ocean [47]. Furthermore, MG III Euryarchaeota were first identified in the deep Northeast Pacific [48], and subsequently in several other aphotic depths in the Mediterranean Sea [15,49], Antarctic Polar Front [50], and Arctic Ocean [47], suggesting that MG III Euryarchaeota are denizens of the deep sea. Although MG I Crenarchaeota (*i.e.*, Thaumarchaeota) and MG II Euryarchaeota are the two most renowned marine Archaea by abundance [49], MG III Euryarchaeota would be a noteworthy archaeal lineage dominant in the deep, consistent with our results (Figure 1B) and those of the deep Arctic Ocean [47].

Water masses at different pelagic depths varied in several physicochemical properties (*e.g.*, temperature, salinity, and nutrient availability), and thus were presumed to act as dispersal barriers for marine prokaryotes [51,52]. Because the SCS is geomorphologically a deep basin, strong stratification and constant ventilation among stratified waters [18] create particular niches for different prokaryotes, which may account for detection of depth-specific OTUs in the SCS water column (Figure 2).

The environment is one of the determinants linked to genomic nucleotide composition [53]. The SCS surface metagenomes had lower genomic %GC values than their deep-sea counterparts (Figure 4), indicating intrinsic differences among collected microbial communities. Low genomic %GC values were prevalent in ocean surface prokaryotes [14,54] and are ascribed to abiotic factors such as nitrogen limitation [55]. As the sea surface environment experiences oxidative stress and solar radiation that can cause DNA mutations, both the inherent AT-biased mutations [56,57] and inadequate repair systems [58-60] are potential factors resulting in low %GC values at ocean surfaces. For the deep SCS, the 3000-m metagenome had two peaks, at 40 and 60 %GC (Figure 4), suggesting parallel abundances of two populations with differing %GC values.

At 10 m in the SCS, cobalamin synthesis protein (COG1429) was prominently enriched in comparison to both deeper SCS waters and other ocean surfaces (Figure 5A). Cobalamin (vitamin B_12_) is heterogeneously distributed (at picomolar concentrations) in the ocean [61,62] and is essential for the synthesis of several enzymes in prokaryotic metabolic systems; *e.g.*, methionine synthase in amino acid synthesis and methylmalonyl-CoA mutase in the citric acid cycle [63]. As the SCS 10-m metagenome also contained more methionine synthase (COG1410) than other surface-sea metagenomes (Figure 6A), the SCS surface was likely to have a greater demand for cobalamin for amino acid synthesis. This was further supported by enrichment of gene suites involved in cobalamin (adjusted *P* = 2.34 × 10^−90^) and isoleucine synthesis (adjusted *P* = 7.98 × 10^−7^) pathways compared with other sea surfaces in the pathway-centric enrichment analysis. Urease (COG0804), which hydrolyzes urea into carbon dioxide and ammonia, was uniquely overabundant at the SCS surface (*P* = 3.21 × 10^−18^, ranked 18th), suggesting a higher urea supply rate in the SCS than other oceans. In part, this may be associated with anthropogenic inputs from the Pearl River and Hainan Island [64]. Prokaryotes are known to acquire urea as an alternative nitrogen source when ammonia is limited [65], and additional measurements of ammonia concentrations may help to associate the enrichment of urease with SCS hydrological conditions.

Similar to deep-sea whale falls [66] and 4000-m HOT [32] metagenomes, the SCS 1000-m community was enriched with COGs for signal transduction (Figure 5B). Bacteria carrying more signal transduction proteins supposedly have larger genomes [67]; therefore, the average genome sizes of the SCS metagenomes were estimated. The 1000-m sample possessed the largest genome size, 3.95 Mbp (Table 3), which was comparable to the genome of the 3000-m sample (3.42 Mbp) and approximately twice as large as the 10- and 100-m metagenomes. This demonstrated that prokaryotes in the meso- and bathypelagic SCS had larger genomes than those at epipelagic depths. Interestingly, chemotaxis proteins were enriched in the aphotic SCS 1000 m, but only in the photic zone of HOT [5]. Therefore, we inferred that the distribution of chemotaxis proteins was driven by factors other than pelagic depth. Signal transduction and chemotaxis proteins were reportedly more common in copiotrophic bacterial genomes [68], suggesting a higher abundance of copiotrophs at 1000 m, corresponding to the depth at which most nutrients reached plateau concentrations (Additional file 1: Figure S8).

In comparison to both shallower SCS depths and other deep oceans, the bathypelagic SCS were especially enriched with metagenomes with plasmid replication initiation functions (COG5527), emphasizing the importance of plasmid genes (usually associated with environmental adaptation) in the 3000-m assemblage. Exonuclease V (COG0507) participates in various DNA-processing pathways (*e.g.*, recombination, repair, and digestion). Its enrichment may be associated with DNA digestion for protecting hosts from foreign DNA, corresponding to the enrichment of transposase in global deep-sea metagenomes (Additional file 1: Table S7) and the supposedly higher frequency of horizontal gene transfer in the deep ocean [5].

As the SCS metagenomes underwent multiple displacement amplification (MDA) to obtain enough DNA for pyrosequencing, those differentially enriched metabolisms that are less correlated with local environmental characteristics might have resulted from the bias introduced by MDA, which was not used in metagenomic studies of other oceans. With respect to the bias of MDA, Kim and Bae [69] demonstrated that single-stranded DNA viruses were preferentially amplified by MDA. This bias in viral DNA amplification could be reduced by using different methods, such as converting viral DNA into double-stranded DNA prior to amplification [70]. To date, most discussion about the bias of MDA has focused on the bacterial community composition [71] and viral metagenomes [72], but the bias towards bacterial metabolic potentials is not fully understood.

## Conclusions

This work presented herein contributes to the knowledge of the microbe-stratified interior of the SCS. The use of 16S rRNA tag sequencing demonstrated the prevalence of Alpha- and Gammaproteobacteria throughout the water column, and the existence of deep-sea Cyanobacteria. Furthermore, MG II and MG III Euryarchaeota were two major Archaea in the surface and bottom SCS, respectively. In addition to the contrasting %GC and average genome size of shallow (10- and 100-m) and deep (1000- and 3000-m) SCS metagenomes, based on embedded metabolism, there was vertical zonation at various pelagic depths. Comparing global sea-surface and deep-sea metagenomes revealed functional preferences delineating epi- and bathypelagic communities. To the best of our knowledge, this is the first attempt to apply community genomics to the SCS, investigating prokaryotic diversity and metabolic potentials among the stratified pelagic zones. The SCS is located between the two most important heat “engines” of the global climate (the Tibetan Plateau and the western Pacific warm pool) and is irregularly subjected to physical forces from the Southeast Asian monsoon, typhoons, strong internal waves, and El Niño-Southern Oscillation, making the SEATS station a unique, ocean time-series study site sensitive to climate changes. Therefore, microbial community variation associated with climatic disturbances (*e.g.*, monsoon and typhoon) and current intrusion (*e.g.*, the Kuroshio Current) is of particular interest for future work in the SCS, which will help to elucidate the ecological interactions between marine microbes and environmental variations.

## Methods

### Sampling site and procedures

Seawater samples were collected during a cruise of the R/V *Ocean Research I* (Cruise 845) of the Oceanography Institute of National Taiwan University during October 21–30, 2007. Our sampling site, the SEATS station, is located at 18°15′N and 115°30′E. We used a rosette multi-bottle array (Model 1015, General Oceanics Inc., Miami, FL, USA) carrying a conductivity-temperature-depth profiler (Sea-Bird Electronics Inc., Bellevue, WA, USA) and 10–12 of the 20-L Go-Flo bottles to collect 140 L of seawater at 10, 100, and 3000 m, and an 80-L bottle at 1000 m (Additional file 1: Table S1). All samples were stored at −20°C in the dark and directly transported to the laboratory by low-temperature delivery as soon as R/V *Ocean Research I* landed, where filtration for microbes was immediately performed. Seawater (500 L in total) was pre-filtered through a 10-μm pore filter (Nitex nylon net, Wildlife Supply Co., Yulee, FL, USA) and sequentially filtered with 0.22-μm membrane filters using a Pellicon cassette tangential flow filtration system with a peristaltic pump (Model XX80EL000, Millipore Corp., Billerica, MA, USA) to collect the retentate. The latter was then concentrated onto a 0.2-μm cellulose acetate filter (Advantec, Japan) using a chemical duty vacuum pump at 80–100 psi (Model WP6111560, Millipore Corp.).

### DNA extraction

Extraction of DNA was performed using the cetyltrimethylammonium bromide method [73] with the following modifications. The retentate on the 0.2-μm cellulose acetate filters was washed with 567 μL of TE buffer (10 mM Tris–HCl at pH 7.5 and 1 mM EDTA at pH 8.0) and the suspension was placed in 1.5-mL microtubes. Sodium dodecyl sulfate (30 μL; 10%) and RNase A (5 μL; 100 mg/mL) was added to each tube and then incubated at 37°C for 1 h. Isolated DNA pellets were air-dried, re-suspended in 10X diluted TE buffer, and stored at −20°C.

### Enumeration of microbes

As bacteria are the most abundant microbes in the ocean, we used bacterial abundance (0.22–10 μm in size) as a proxy for microbial abundance in the SCS. Enumeration was conducted as follows. Concentrated samples were diluted with appropriate volumes of seawater (≥1 mL), and 0.8-mL aliquots of the diluted samples were collected onto 0.02-μm pore membrane filters overlaid on pre-washed 0.45-μm pore membrane filters. Filtration pressure was consistently <15 kPa. The staining solution contained 10% SYBR Gold (ddH_2_O:SYBR Gold = 9:1; Invitrogen, Carlsbad, CA, USA) and antifade mounting medium (2 μL of 10% p-phenylenediamine dihydrochloride in 198 μL of glycerol:PBS (1:1,v/v) solution). The 0.02-μm pore membrane filters with microbes were dried in a laminar flow hood, placed on Petri dishes pre-loaded with 80 μL of SYBR Gold working solution, stained for 15–20 min in the dark, and fixed with antifade mounting medium. Filters were examined with an epifluorescence microscope (Eclipse 90i, Nikon Corp., Japan). Twenty distinct fields were counted on each filter using the imaging software NIS-Elements (Nikon Corp.).

### 16S rRNA tag sequencing sample preparation and pyrosequencing

The hypervariable V6 region of 16S rRNA genes (abbreviated as V6) was adopted to probe bacterial and archaeal community composition. Tag sequencing samples were prepared as described [74], with primers 967 F (5′-CAACGCGAAGAACCTTACC-3′) and 1046R (5′-CGACAGCCATGCANCACCT-3′) for bacteria, and 958arcF (5′-AATTGGANTCAACGCCGG-3′) and 1048arcR (5′-CGRCGGCCATGCACCWC-3′) for archaea. Approximately 200 ng of each tagged V6 library was pooled and sent to Mission Biotech Corp. (Taipei, Taiwan) for sequencing with the Genome Sequencer FLX System (Roche 454 Life Sciences, Branford, CT, USA). At that facility, the software GS Run Processor (v2.5, Roche 454 Life Sciences) was applied for read quality control with default settings. Reads were removed if they were: (1) shorter than 75 bp, (2) longer than 115 bp, or (3) contained any mismatch in primers. The remaining V6 reads were sorted into different samples by tag (barcode) sequences using an in-house program (http://tanglab.csie.org/sorter/).

### Taxonomic identification

All bacterial and archaeal V6 amplicon reads were processed by the SILVA-ngs pipeline [27] for taxonomic identification, and the process is summarized below.

Each V6 amplicon read was aligned using the SILVA Incremental Aligner (SINA, v1.2.10) [75] against the SILVA SSU rRNA SEED and quality controlled by removing reads (1) with <50 aligned nucleotides, (2) with >2% ambiguities, or (3) with >2% homopolymers [27]. Potentially contaminated samples and artifacts (*i.e.*, reads of <50% alignment identity or a <40 alignment score reported by SINA) were also excluded, resulting in 38,360 bacterial and 5182 archaeal V6 reads for downstream analysis.

On a per-sample basis, identical reads were identified (*i.e.*, dereplication) and unique reads were used for operational taxonomic unit (OTU) generation. Dereplication and OTU generation were performed using CD-HIT-EST (v3.1.2) [76] running in accurate mode, ignoring overhangs, and applying identity thresholds of 1.00 and 0.98, respectively. The representative V6 read of each OTU was classified through a nucleotide BLAST search against the non-redundant version of the SILVA SSU Ref dataset (release 115) using BLASTn with standard settings [77]. The taxonomic classification of representatives was mapped onto all reads in the respective OTU. Reads without any BLAST hit or with weak BLAST hits, where the function “(% sequence identity + % alignment coverage)/2” did not exceed the value of 93, remained unclassified and were assigned to “No Relative”.

After SILVA-ngs analysis, the largest OTU of Cyanobacteria (containing 287 reads) detected at 3000 m were manually checked again using the web-BLASTn against the NCBI non-redundant nucleotide database.

### Community diversity indices estimation

The OTUs defined by the SILVA-ngs pipeline (at 98% similarity level) were analyzed using Mothur (v1.29.2) [28] to calculate the Shannon index, Chao 1 estimator, Simpson index, Good’s coverage, and rarefaction curve, which were averaged from the resampling process with 1000 iterations. In bacterial 16S rRNA tag sequencing samples, four OTUs of archaea (6 reads) and 38 OTUs of chloroplast (80 reads) were excluded from diversity indices calculation. The OTU profiles were generated using R (http://www.r-project.org) based on the relative abundance of all OTUs per sample.

### Bacterial 16S rRNA tag sequencing samples from other oceans and related analysis

By considering the availability of bacterial 16S rRNA tag sequencing samples (on V6 hypervariable region) covering the entire water column (from epipelagic, mesopelagic, to bathypelagic depth), we selected three oceanic regions for comparison: the Azores (37°20′N, 18°53′W), the Mediterranean Sea (40°39′N, 2°51′E), and HOT (22°45′N, 158°0′W). The sampling depths are listed below; Azores: 0, 100, 1200, and 3660 m; the Mediterranean Sea: 5, 500, and 2000 m; HOT: 10, 100, 1000, and 3000 m. A detailed sample list is available in Additional file 1: Table S9. All amplicon datasets were downloaded from the Visualization and Analysis of Microbial Population Structures database (https://vamps.mbl.edu) and analyzed using the same methods as those applied on the SCS sample (*i.e.*, using SILVA-ngs to generate OTUs and identify taxonomy; using Mothur to estimate diversity indices by resampling with 1000 iterations).

Amplicon read counts of all taxa were total-sum scaled per sample before hierarchical clustering and non-metric multidimensional scaling (nMDS) analysis. Hierarchical clustering was performed in R using complete linkage and nMDS analysis was carried out using the R package vegan [78] at class and genus level separately. The Bray-Curtis distance was applied in both analyses.

### Metagenome sequencing

The total DNA of each sample from the SEATS water column was amplified with a multiple displacement amplification Kit (Genomophi V2 DNA Amplification Kit, GE Healthcare Life Sciences, Piscataway, NJ, USA), according to the manufacturer’s instruction. Small oligonucleotides in the samples were removed by centrifugation using microspin G-50 columns (GE Healthcare Life Sciences). Approximately 5 μg DNA of each sample was sequenced with the Genome Sequencer FLX System (Roche 454 Life Sciences) at Mission Biotech Corp. The default settings in GS Run Processor (v2.5, Roche 454 Life Sciences) were used for read quality control. High-quality reads were subsequently assembled using GS *de novo* Assembler (v1.1.02, Roche 454 Life Sciences) with a 40-bp minimum overlap and 99% consensus. Open reading frames were predicted from assembled contigs by using MetaGeneMark (http://exon.gatech.edu).

### Bioinformatics analysis on metagenomes

Metagenomic GC content (%GC) was calculated from contigs using the R package SeqinR [79]. Functional annotation of putative ORFs was assigned to the best match in the eggNOG (v3.0) database [80] through BLASTp (*e*-value ≤10^−5^). To identify particular functions that were statistically abundant or deficient in one metagenome compared with the other, functional enrichment analysis (*i.e.*, two-group comparison) was performed on the Clusters of Orthologous Groups (COGs) family frequencies using the R package ShotgunFunctionalizeR [32]. This analysis normalized gene family frequencies using a generalized linear model with Poisson canonical logarithmic link function and determined the significance (*P*-value) using a binomial method, with the Benjamini-Hochberg false-discovery rate correction to adjust q-values for multiple testing. In this research, COG gene family frequency was quantified as the number of reads mapped to ORFs in each metagenome. Read mapping was performed using MegaBLAST to search ORF nucleotide sequence against reads per sample. Every read was mapped to a single ORF of the highest bit score if the MegaBLAST alignment had an identity ≥90% and *e*-value ≤10^−5^. Every ORF had a minimum read number of one. Poisson regression was also performed on COG gene family frequency versus log-transformed depths using ShotgunFunctionalizeR. Average genome size of metagenome was estimated by GAAS [81].

### Other oceanic metagenomes included in comparative analysis

The HF10 metagenome taken from HOT [5] and seven metagenomes from the oceans (GS001c and GS018 from the Atlantic Ocean; GS026, GS037, and GS047 from the Pacific Ocean; and GS113 and GS123 from the Indian Ocean) collected by the Global Ocean Sampling (GOS) expedition [4] were selected to represent global sea-surface datasets. Deep-sea metagenomes including Km3 [15] and matapan [82] from the deep Mediterranean Sea, PRT from the Puerto Rico Trench [17], and HF4000 from HOT were compiled to represent deep-sea datasets. All metagenomes were downloaded from the CAMERA website (http://camera.calit2.net) except Km3, matapan, and PRT, which were directly obtained from the authors. The COG frequencies of matapan and PRT metagenomes were quantified using the read number of ORFs by the same approach (MegaBLAST) as the SCS metagenomes. Read mapping of the GOS metagenomes were performed using tBLASTn to search ORF peptide sequence against reads per sample and quantified with the same criteria as the SCS metagenome. The COG frequencies of HF10, HF4000, and Km3 were quantified by the number of ORFs, because these metagenomes were derived directly from fosmid library sequencing without assembly.

### Availability of supporting data

Bacterial and archaeal 16S rRNA tag sequencing reads and metagenomes in the SCS have been deposited in the NCBI Sequence Read Archive [SRA048273].

## Additional file

Additional file 1:
**Supplementary Information.** This file contains supplementary details to Supplementary Methods, Results, Figures, and Tables.

## Abbreviations

COGs: Clusters of Orthologous Groups
GOS: Global Ocean Sampling
HOT: Hawaii Ocean Time-series
MG: Marine Group
OTU: Operational Taxonomic Unit
PRT: Puerto Rico Trench
SCS: South China Sea
SEATS: South East Asia Time-series Study
