# Supplementary material for: Prokaryotic assemblages and metagenomes in pelagic zones of the South China Sea
Source: BMC Genomics. 2015 Mar 20;16(1):219. doi: 10.1186/s12864-015-1434-3 (PMC4373125; doi:10.1186/s12864-015-1434-3)
Supplement: Additional file 1: — Supplementary Information. This file contains supplementary details to Supplementary Methods, Results, Figures, and Tables. [file 12864_2015_1434_MOESM1_ESM.pdf]

Additional File 1 – Supplementary Information

Prokaryotic assemblages and metagenomes in pelagic zones of the South China Sea

Ching-Hung Tseng, Pei-Wen Chiang, Hung-Chun Lai, Fuh-Kwo Shiah, Ting-Chang Hsu, Yi-Lung Chen, Liang-Saw Wen, Chun-Mao Tseng, Wung-Yang Shieh, Isaam Saeed, Saman Halgamuge and Sen-Lin Tang

Methods

Sampling and DNA extraction for DGGE analysis
PCR-DGGE analysis

Results

Hydrography of the South China Sea
Poisson regression analysis

Figure

Figure S1. PCR-DGGE analysis of the 16S rRNA genes from the SCS
Figure S2. Water temperature, salinity, and density profile in the SCS
Figure S3. The temperature-salinity diagram in the SCS
Figure S4. Rarefaction curves of (A) bacterial and (B) archaeal community in the SCS
Figure S5. Bacterial OTU profiles in the SCS
Figure S6. Archaeal OTU profiles in the SCS
Figure S7. nMDS analysis of bacterial communities in the SCS and other oceans
Figure S8. Vertical profile of oceanographic parameters in the SCS
Figure S9. Top-10 COGs of decreasing abundance with increasing depth in the SCS
Figure S10. Top-10 COGs of increasing abundance with increasing depth in the SCS
Figure S11. Top-10 globally enriched functions in ocean surfaces versus deep oceans
Figure S12. Top-10 globally enriched functions in deep oceans versus ocean surfaces
Figure S13. Phylogenetic tree of Betaproteobacteria V6 amplicon reads

Table

Table S1. Oceanographic data measured in the SCS during Cruise 845
Table S2. Bacterial and archaeal diversity indices based on 16S rRNA gene libraries of the SEATS station
Table S3. Bacterial diversity indices based on 16S rRNA gene libraries of the SEATS station and other oceans
Table S4. Reciprocal tBLASTx analysis results
Table S5. Pearson's correlation coefficients among oceanographic parameters
Table S6. Top-20 globally enriched functions in ocean surfaces versus deep oceans
Table S7. Top-20 globally enriched functions in deep oceans versus ocean surfaces
Table S8. Bacterial and archaeal primer coverages
Table S9. Bacterial 16S rRNA V6 amplicon libraries from other oceans

Methods

Sampling and DNA extraction for DGGE analysis

Seawater samples were collected at the SEATS station (18°15'N, 115°30'E) on October 20 and 21, 2006. Twenty liter Go-Flo sampling bottles with CTD rosettes were deployed for water collection at 15 different depths covering epi- to bathypelagic zones (*i.e.*, 10, 20, 30, 40, 50, 60, 80, 100, 300, 400, 500, 1000, 1200, 1500, and 2000 m). For each depth, 700 ml seawater was collected and stored in 1-liter plastic carboy bottles at -80 °C until DNA extraction. In laboratory, the seawater samples were melted on ice and filtered for bacterioplanktons using cellulose acetate membranes of 0.2 µm pore size (ADVANTEC). The membranes were removed by sterile forceps to clean centrifuge tubes and washed with TE buffer (50 mM Tris-HCl and 1 mM EDTA at pH 8.0). The solution was collected in a microtube and used for DNA extraction as previously described [1]. The DNA pellet was then resolved in sterilized water, and the DNA solution was aliquoted into smaller volumes for storage at -20 °C.

PCR-DGGE analysis

To amplify the 16S rRNA gene for DGGE, a PCR was conducted with a pair of universal primers, 341F with GC clamp (5'-CGC-CCG-CCG-CGC-GCG-GCG-GGC-GGG-GCG-GGG-GCA-CGG-GGG-GCC-TAC-GGG-AGG-CAG-CAG-3') and 907R (5'-CCG-TCA-ATT-CMT-TTG-AGT-TT-3'). The total volume was 50 µl, including 200 µM dNTP, 0.5 µM of each primer, 1.5 µl (1.5 U) of *Taq* enzyme (Finnzymes, DyNAzyme EXT), 1.5 mM of $MgCl_2$, 5 µl of 10 X PCR buffer (Finnzymes), and 30 ng of template DNA. The amplification program was conducted using a PxE Thermal Cycler (Thermo Electron Corp.). The first cycle was initiated at 94°C for 5 min, and followed by 30 cycles. Each cycle was 94°C for 30 sec, 47°C for 30 sec, and 72°C for 1 min. The last cycle was 72°C for 10 min before cooling at 4°C. The PCR products were checked using electrophoresis and visualized by a UV *trans*-illuminator, ImageQuant 300 (GE Healthcare). The PCR product was resolved by DGGE using the DCode gel system (Bio-Rad). The acrylamide concentration was 7% (bis-acrylamide gel stock solution, 37.5:1). The running buffer was 1X Tris-actate-EDTA and the denaturing gradient was set from 30-45%. Electrophoresis was conducted at 70 Volts and 60°C for 13 h. The gel was visualized using silver staining [2].

To verify the identity of bands in the gel, selected bands were firstly cut out and resolved in small volumes of Milli-Q water. The DNA fragments were amplified by PCR using the primers 341F without GC clamp and 907R, and the PCR product was confirmed by 1.5% agarose electrophoresis. If the product contained non-specific DNA, the desired DNA band would be cut out from the agarose gel for purification again using the QIAEXII gel extraction kit (QIAGEN), and sent to sequencing at Mission Biotech Corp. (Taipei,

Taiwan) for confirmation. A total of 139 bacterial rDNA sequences from the DGGE were identified, among which 13 sequences were obtained by the clone-library because of the unsuccessful purification of methods mentioned above. Sequences longer than 150 base pairs and best matched to 16S rDNA homologs in the NCBI nucleotide database with ≥95% identity have been deposited in the NCBI GenBank [HQ879850-HQ879969].

Results

Hydrography of the South China Sea

The temperature profile of the SEATS water column was continuously stratified (Additional File 1, Figure S2). The thermocline, which separates the upper mixed layer from the calm deep water, was approximately between 70 and 200 m. The temperature was nearly 28°C at the upper mixed layer and decreased as the depth increased, whereas the potential density showed a contrary profile along depth. The salinity was about 33.6 psu at the sea surface, increased with increasing depth, peaked to 34.6 psu at 130 m, decreased slightly to a local minimum 34.4 psu between 350 and 430 m, and increased with increasing depth again. The salinity was stable below 1000 m (34.5 psu at 1000 m; 34.6 psu at 3000 m).

The temperature-salinity diagram indicated that three water masses existed in the sampling site (Additional File 1, Figure S3), corresponding to previous reports [3-5]. The northern SCS surface water is influenced by both the freshwater input from the Pearl River in China and the Kuroshio intrusion, so it has lower salinity than the Kuroshio and Pacific water. The SCS intermediate water has similar characteristics with the North Pacific Intermediate Water [5]. The cold deep water is a mixture of Circumpolar Deep Water and Pacific Subarctic Intermediate Water [6, 7].

Several nutrients were measured during the sampling cruise (Additional File 1, Table S1). The concentration of dissolved oxygen was highest in the surface water (about 200 μM), decreased with increasing depth, and rose to about 110 μM at 3000 m. The concentrations of nitrite, nitrate, dissolved inorganic phosphate, and silicate increased with increasing depth. The concentration of chlorophyll-*a* in the SCS surface (0.16 $\mu g\ l^{-1}$) and dissolved inorganic carbon was approximately 2000 $\mu mol\ kg^{-1}$. The pH values in SCS decreased with increasing depth. Consistency of our measurements to previous reports [8-10] demonstrated a relative stable nature of these nutrient concentrations in the SCS.

Poisson regression analysis

Most of the top-10 significant COGs (Pearson correlation, P-value ≤ 0.05) showing negative correlation to the depth (*i.e.*, the deeper, the less) enriched at 10 m, such as Mg-chelatase (COG1239), Glucose-6-P dehydrogenase (COG3429), cobalamin biosynthesis protein CobN (COG1429), and photolyase (COG0415) (Additional File 1, Figure S10). Similarly, COGs enriched at 1000 and 3000 m were more abundant at deeper depths, including iron transporter (COG1629), histidine kinase (COG0642), chemotaxis protein (COG0840), and plasmid replication initiation protein (COG5527 and COG5655). Transcription regulator (COG0583) and efflux pump (COG3696) were likely also metabolisms specific to the deep SCS community (Additional File 1, Figure S11).

Figure

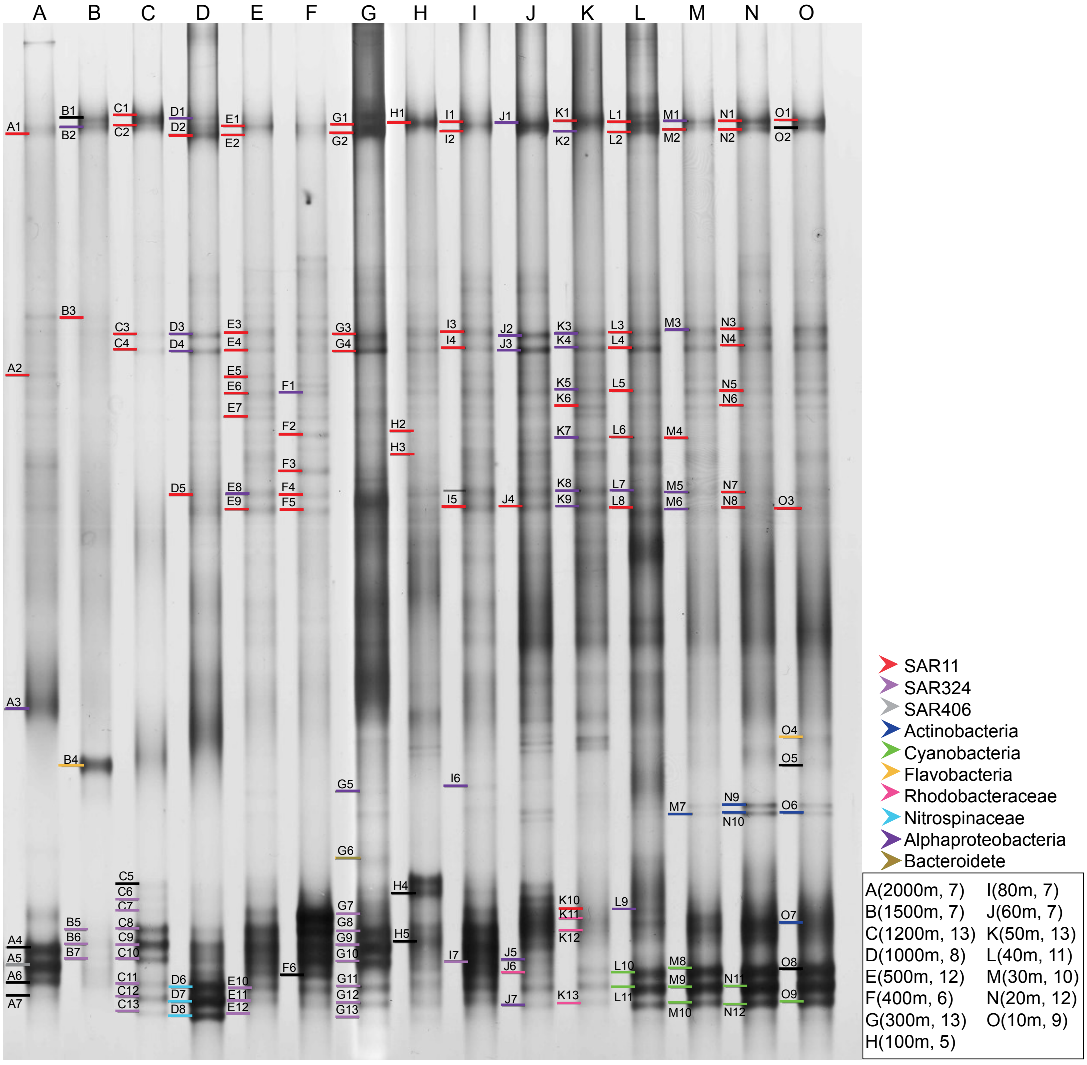

Figure S1. PCR-DGGE analysis of the 16S rRNA genes in the SCS. Color lines represent different bacterial taxonomic groups. These groups include SAR11 (red line), SAR324 (light purple line), SAR406 (gray line), Actinobacteria (blue line), Cyanobacteria (green line), Flavobacteria (yellow line), Rhodobacteraceae (pink line), Nitrospinaceae (light blue line), Alphaproteobacteria (purple line), and Bacteriodetes (brown line). Black lines are sequences with <95% alignment identity to the best match in the NCBI nucleotide database. Lanes A to O represent samples of different depths, which are shown in figure and followed by the number of bands that were cut and sequenced.

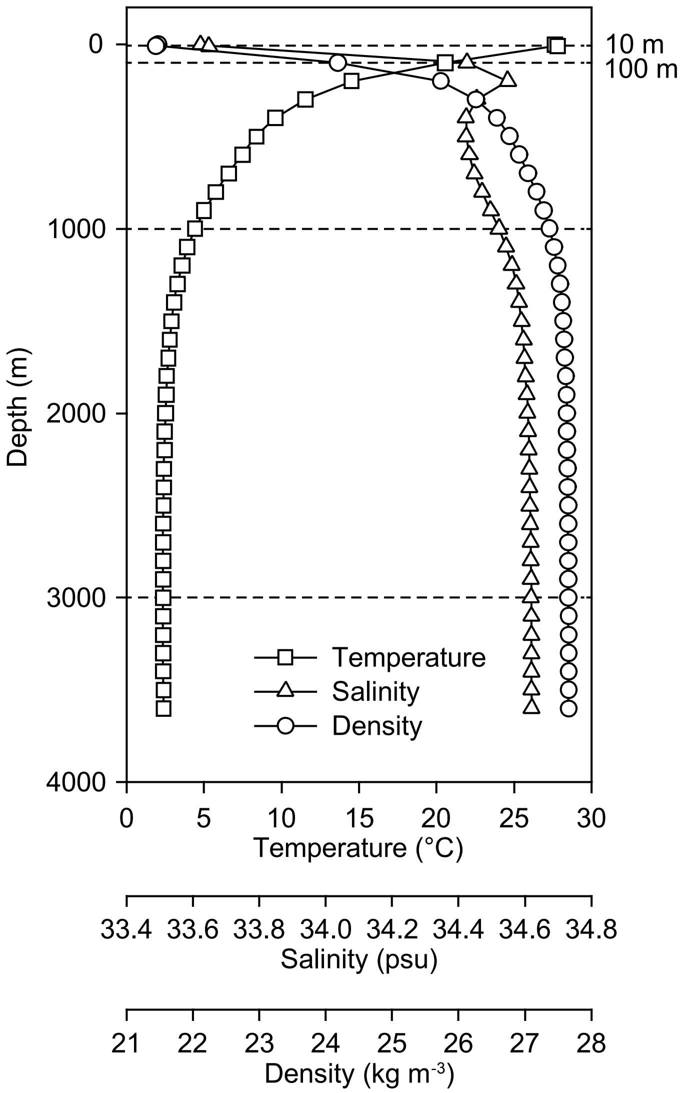

Figure S2. Water temperature, salinity, and density profile in the SCS

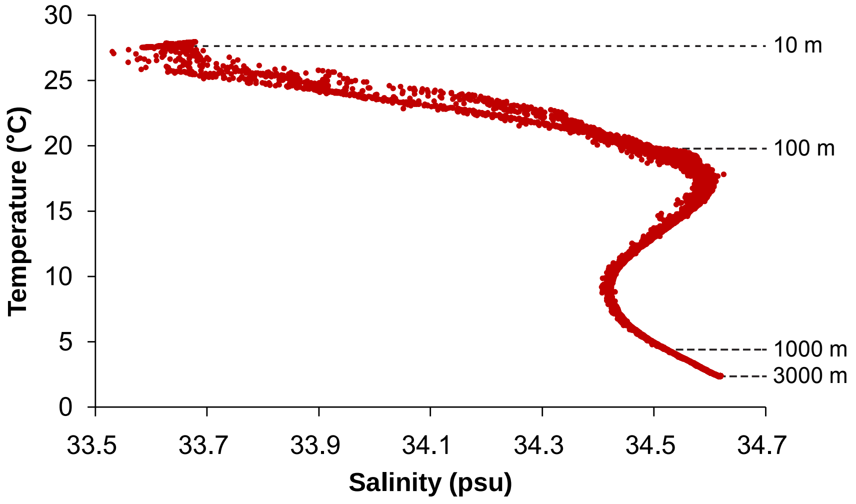

Figure S3. Temperature-Salinity diagram in the SCS. Dashed lines indicate four sampling depths.

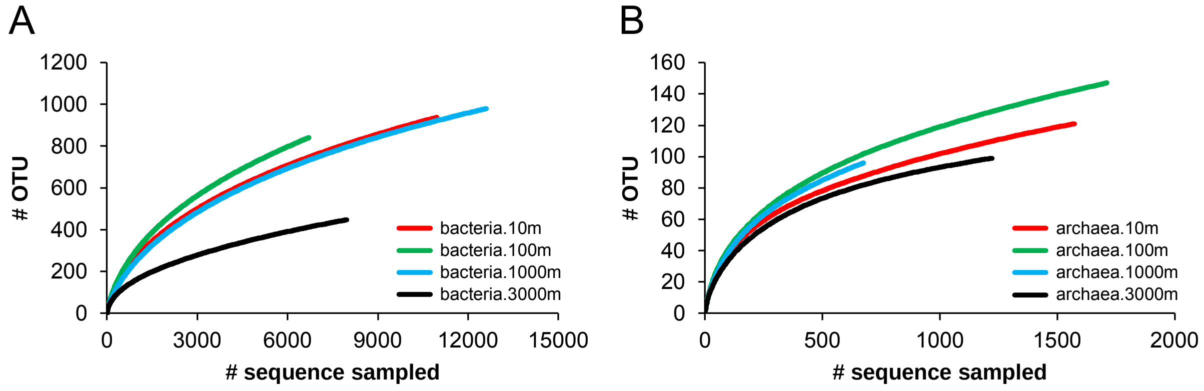

Figure S4. Rarefaction curves of (A) bacterial and (B) archaeal community in the SCS.

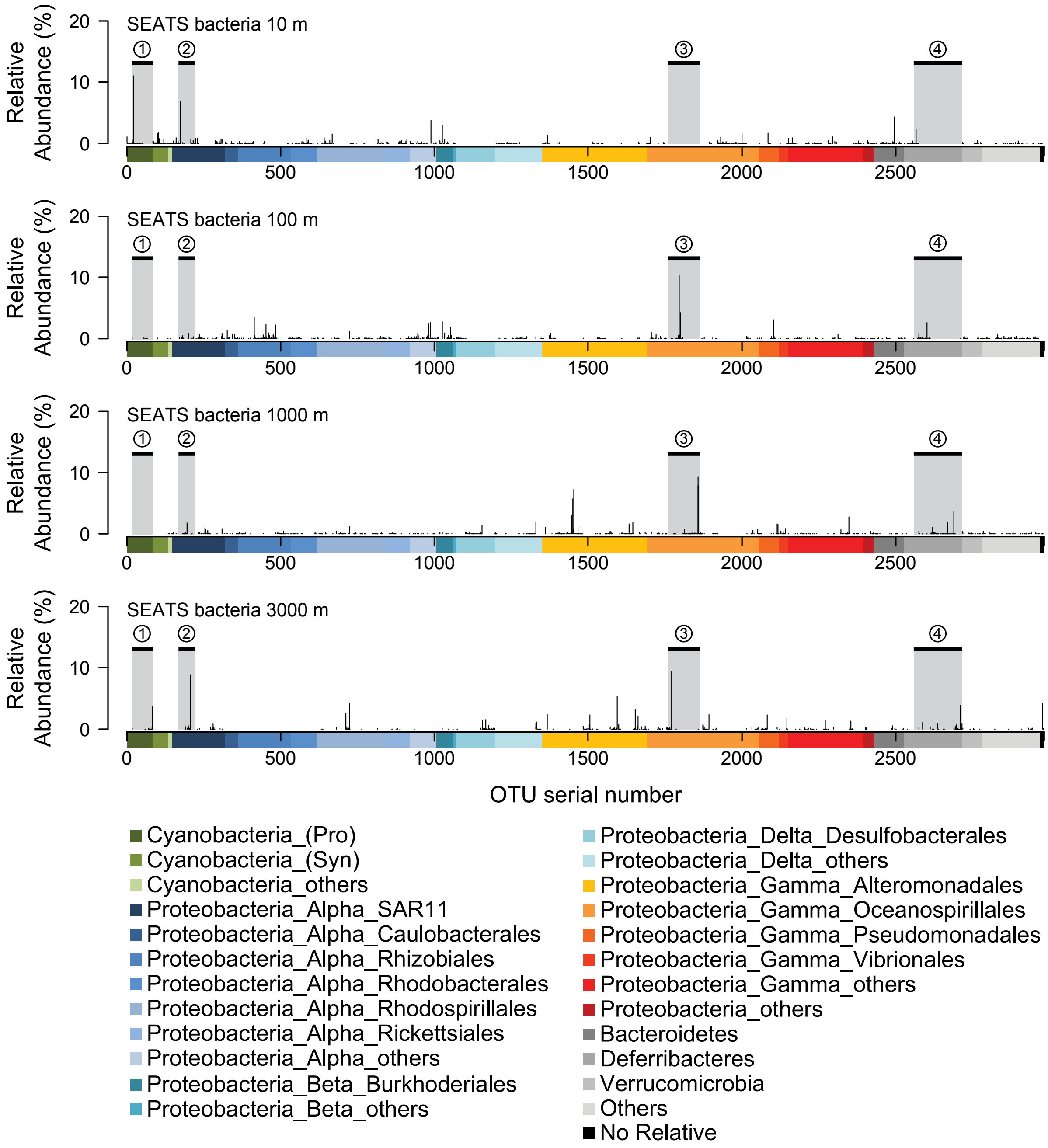

Figure S5. Bacterial OTU profiles in the SCS. The x-axis represents 2982 OTUs from the whole bacterial dataset. Different taxa are colored accordingly and named in the format of phylum or phylum_class_order for clarity. Taxon names deeper than order level are listed in parenthesis. Bacterial phyla with <1% relative abundance in average are grouped into "Others". Bacterial OTUs of interest are shaded in gray with numbers labeled in the figure. Abbreviations: Pro, *Prochlorococcus*; Syn, *Synechococcus*; Alpha, Alphaproteobacteria; Beta, Betaproteobacteria; Gamma, Gammaproteobacteria; Delta, Deltaproteobacteria.

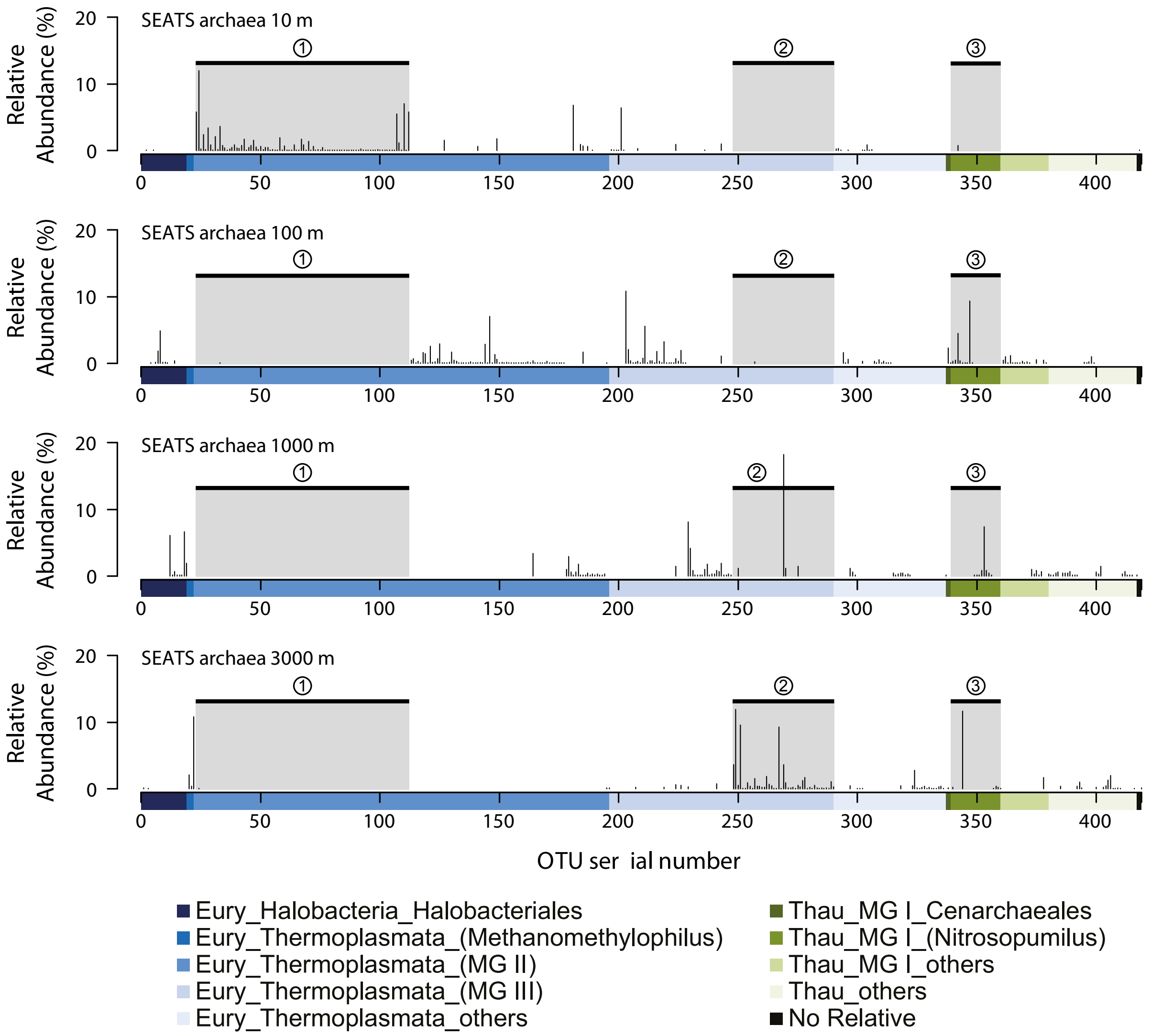

Figure S6. Archaeal OTU profiles in the SCS. The x-axis represents 419 OTUs from the whole archaeal dataset. Different taxa are colored accordingly and named in the format of phylum or phylum_class_order for clarity. Taxon names deeper than order level are listed in parenthesis. Archaeal OTUs of interest are shaded in gray with numbers labeled in the figure. Abbreviations: Eury, Euryarchaeota; Thau, Thaumarchaeota; MG, Marine Group.

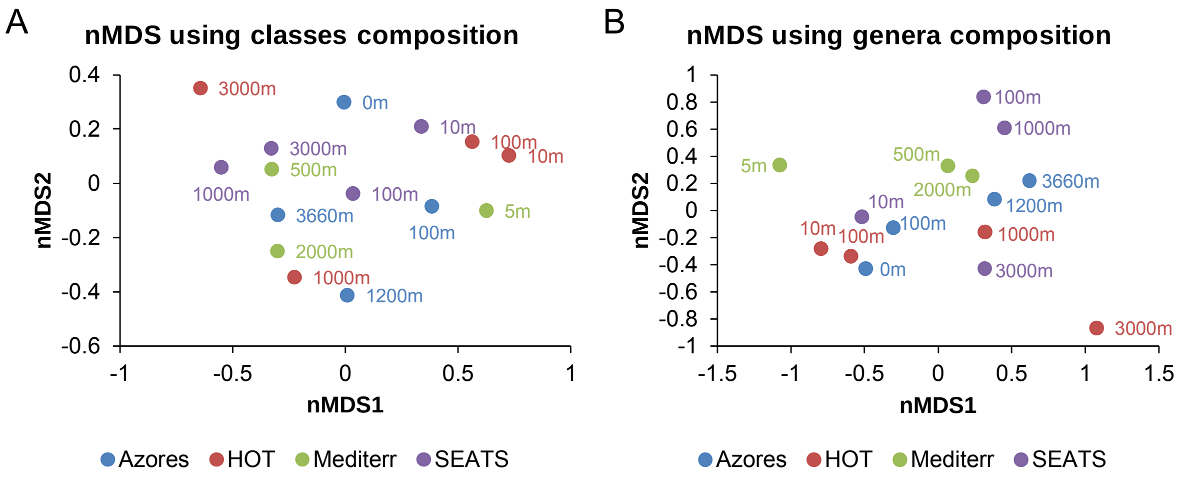

Figure S7. nMDS analysis of bacterial communities in the SCS and other oceans. nMDS ordination results of bacterial community at (A) class and (B) genus level, containing 94 classes and 875 genera, respectively, were performed. Sampling depths are labeled next to each data points.

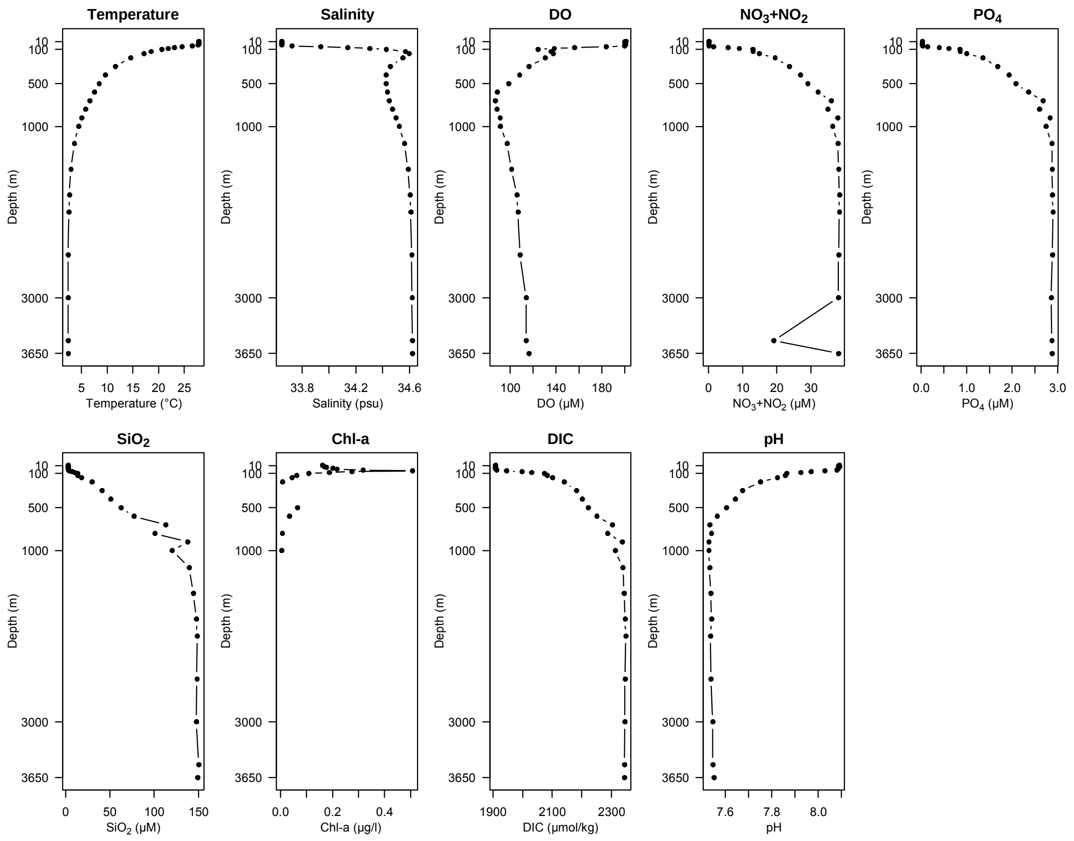

Figure S8. Vertical profile of oceanographic parameters in the SCS. Data were measured during Cruise 845. Data are values averaged from multiple casts at different depths.

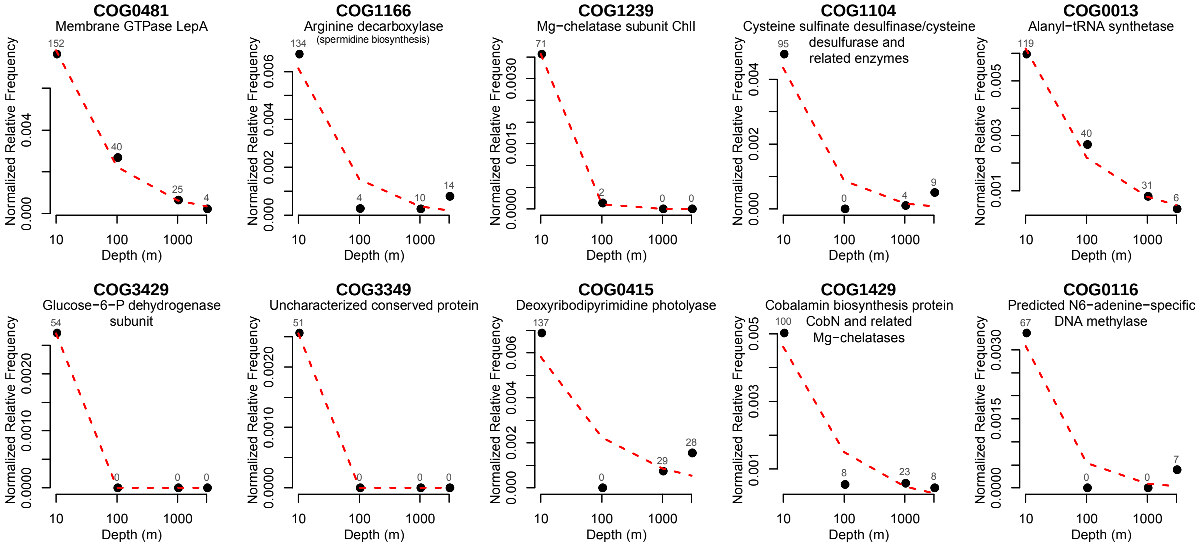

Figure S9. Top-10 COGs of decreasing abundance with increasing depth in the SCS. The original counts of reads are labeled around every data point. Regression lines are shown as dashed lines in red.

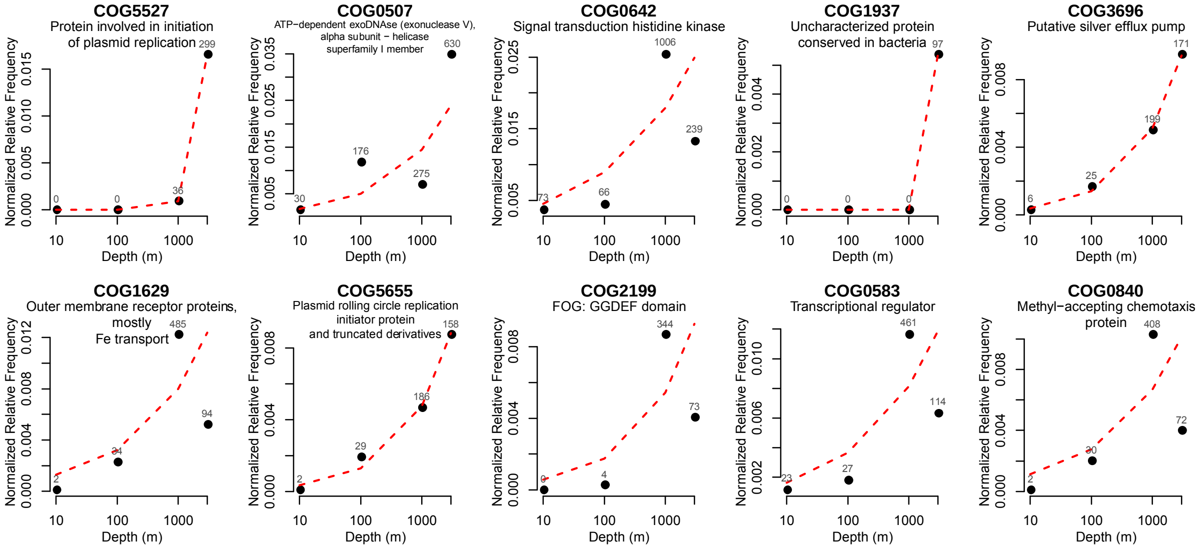

Figure S10. Top-10 COGs of increasing abundance with increasing depth in the SCS. The original counts of reads are labeled around every data point. Regression lines are shown as dashed lines in red.

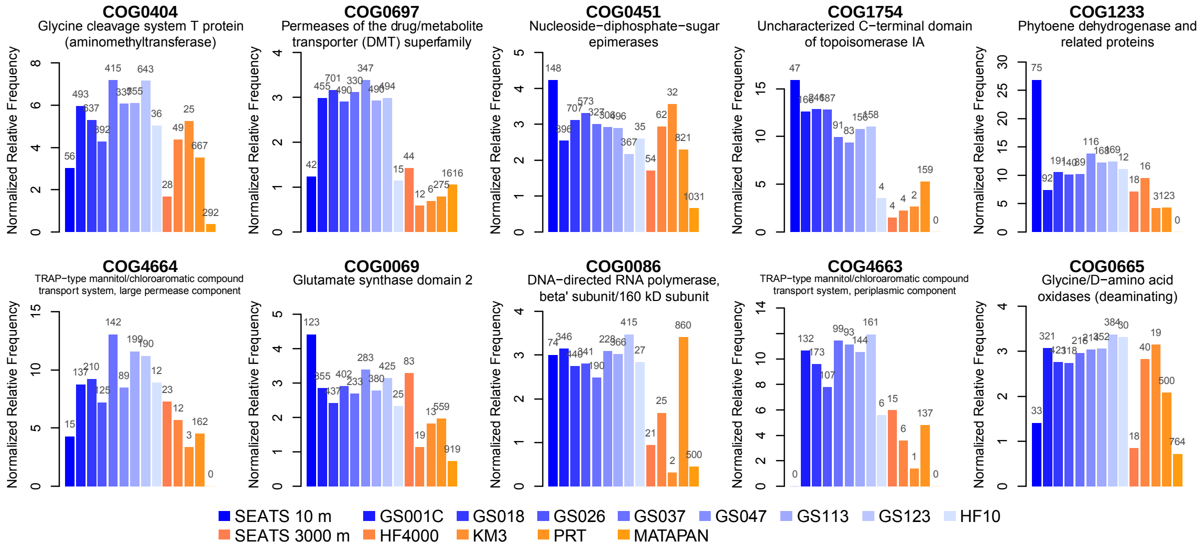

Figure S11. Top-10 globally enriched functions in ocean surfaces versus deep oceans. The original counts are labeled on each bar.

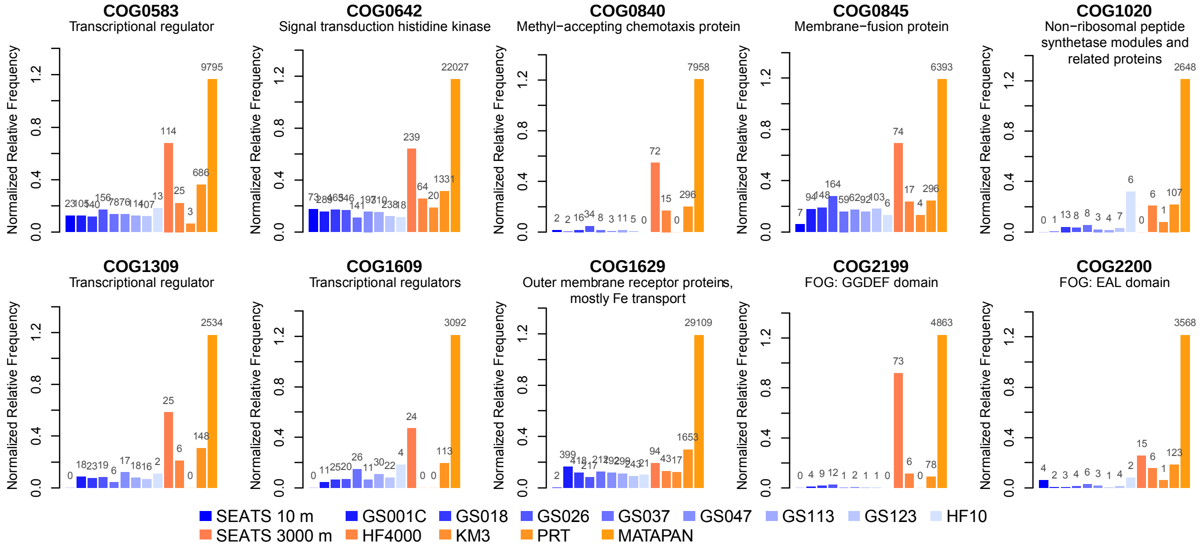

Figure S12. Top-10 globally enriched functions in deep oceans versus ocean surfaces. The original counts are labeled on each bar.

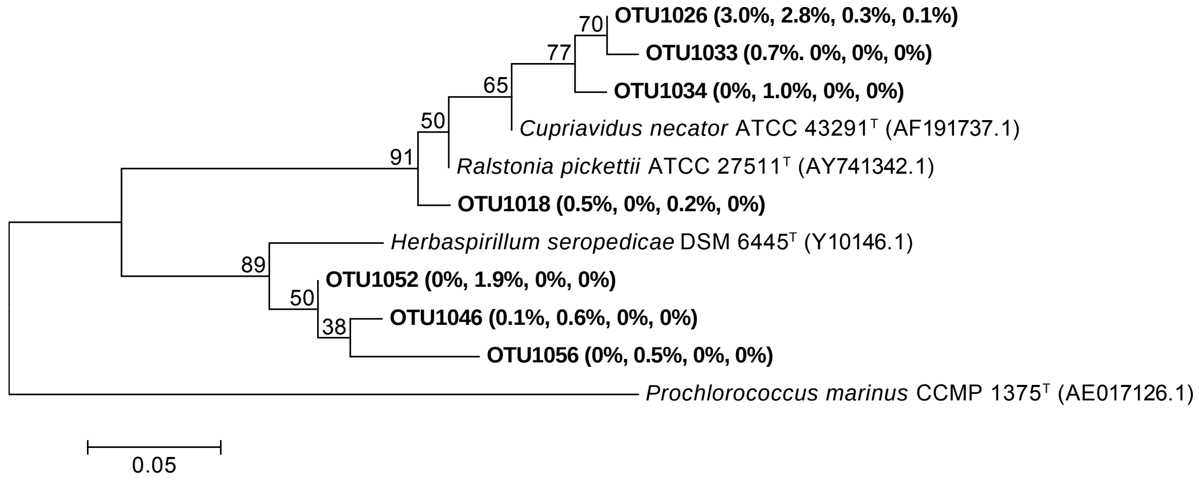

Figure S13. Phylogenetic tree of Betaproteobacteria V6 amplicon reads. OTUs of Betaproteobacteria with >0.45% relative abundance at least in one sample were selected for this analysis. OTU representative V6 amplicon reads and references were aligned by using Muscle (v3.8.31) and trimmed by Gblocks allowing smaller final blocks containing gaps [11]. The maximum likelihood tree was inferred from 103 aligned positions of the 16S rRNA hypervariable V6 region and derived based on the General Time Reversible model using MEGA 6 [12]. Bootstrap values (expressed as percentages of 1000 replicates) are shown at branch points. Scale bar represents 0.05 substitutions per nucleotide position. OTUs in this study are indicated in boldface followed by relative abundances at 10, 100, 1000, and 3000 m depths in parenthesis. Type strains are indicated with a superscript T, followed by the accession number registered in GenBank database in parenthesis. *Prochlorococcus marinus* CCMP 1375^T was used as an outgroup.

Table

Table S1. Oceanographic data measured in the SCS during Cruise 845. Data are the mean ± standard deviation of multiple CTD casts except cell density.

Parameters	10 m	100 m	1000 m	3000 m
Volume filtered (liters)	140	140	80	140
Temperature (˚C)	27.79±0.09	20.56±0.59	4.42±0.02	$2.36\pm2.00\times10^{-3}$
Salinity (psu)	33.65±0.02	34.42±0.05	$34.52\pm2.00\times10^{-3}$	$34.62\pm9.57\times10^{-5}$
Dissolved oxygen (μM)	199.84±0.16	124.02±18.86	91.25±1.28	$113.85\pm1.42\times10^{-4}$
Nitrate, NO_3^- (μM)	0.02±0.03	12.88±1.62	36.29±0.15	37.99±0.10
Nitrite, NO_2^- (μM)	0.00±0.00	0.04±0.01	$1.00\times10^{-3}\pm1.00\times10^{-3}$	$1.00\times10^{-3}\pm2.00\times10^{-3}$
DIP (μM)	$0.03\pm4.00\times10^{-3}$	0.85±0.14	2.74±0.04	$2.86\pm5.00\times10^{-4}$
Silicate, SiO_4^{2-} (μM)	2.64±0.04	13.27±1.53	120.05±1.31	147.55±0.54
Chlorophyll a (μg l^{-1})	0.16±0.02	0.11±ND	4.00×10^{-3}±ND	ND
DIC (μmol kg^{-1})	1907.89±1.80	2073.21±1.13	2312.09±0.59	2344.50±1.30
pH	$8.09\pm1.00\times10^{-3}$	7.86±0.02	$7.53\pm3.00\times10^{-3}$	$7.55\pm3.00\times10^{-3}$
Microbial abundance (cell ml^{-1})	5.04×10^{4}	1.02×10^{5}	5.46×10^{4}	1.97×10^{4}

Abbreviations: DIP, dissolved inorganic phosphate; DIC, dissolved inorganic carbon; ND, not determined.

Table S2. Bacterial and archaeal diversity indices based on 16S rRNA gene libraries of the SEATS station.

Samples[a]	N	# OTU[b]	# Singleton OTU	Shannon	Simpson	Chao 1	Evenness[c]	Richness[d]	Good's coverage[e]
Bac 10m	10964	938	418	4.973	0.025	1490	0.73	103.22	0.962
Bac 100m	6711	841	402	5.124	0.021	1491	0.76	104.79	0.94
Bac 1000m	12612	980	422	4.713	0.03	1493	0.68	102.66	0.967
Bac 3000m	7987	448	211	4.211	0.033	764	0.69	53.81	0.974
Arc 10m	1573	121	46	3.666	0.044	190	0.76	14.08	0.971
Arc 100m	1712	147	57	3.811	0.041	227	0.76	17.32	0.967
Arc 1000m	675	96	39	3.535	0.06	153	0.77	13.43	0.942
Arc 3000m	1222	99	29	3.375	0.063	128	0.73	9.07	0.976

[a]Bac indicates Bacteria whereas Arc indicates Archaea.
[b]OTUs are defined at 98% sequence similarity using 16S rRNA hypervariable V6 region.
[c]Evenness is defined as Shannon/ln(# OTU).
[d]Richness is defined as (# singleton OTU-1)/$\log_{10}$N. The maximum value is (N-1)/$\log_{10}$N.
[e]Good's coverage is defined as 1-(# singleton OTU)/N.

Table S3. Bacterial diversity indices based on 16S rRNA gene libraries of the SEATS station and other oceans.

Samples	N	# OTU[a]	# Singleton OTU	Shannon	Simpson	Chao 1	Evenness[b]	Richness[c]	Good's coverage[d]
Azores 0m	23757	1152	528	4.479	0.039	1888	0.64	120.44	0.978
Azores 100m	20706	1521	724	4.909	0.041	2689	0.67	167.51	0.965
Azores 1200m	20398	1875	686	5.481	0.024	2666	0.73	158.95	0.966
Azores 3660m	25376	2180	1003	5.137	0.036	3531	0.67	227.5	0.961
HOT 10m	21123	791	274	3.904	0.084	1144	0.59	63.12	0.987
HOT 100m	22194	1241	561	4.633	0.043	2217	0.65	128.85	0.975
HOT 1000m	17973	1843	871	5.398	0.018	3085	0.72	204.48	0.952
HOT 3000m	14897	832	332	4.34	0.043	1255	0.65	79.32	0.978
Mediterr 5m	21691	502	176	3.912	0.051	713	0.63	40.36	0.992
Mediterr 500m	12276	1021	343	5.082	0.026	1383	0.73	83.64	0.972
Mediterr 2000m	14303	1499	742	5.166	0.029	2626	0.71	178.32	0.948
SEATS 10m	10964	938	418	4.973	0.025	1490	0.73	103.22	0.962
SEATS 100m	6711	841	402	5.124	0.021	1491	0.76	104.79	0.94
SEATS 1000m	12612	980	422	4.713	0.03	1493	0.68	102.66	0.967
SEATS 3000m	7987	448	211	4.211	0.033	764	0.69	53.81	0.974

[a]OTUs are defined at 98% sequence similarity using 16S rRNA hypervariable V6 region.
[b]Evenness is defined as Shannon/ln(# OTU).
[c]Richness is defined as (# singleton OTU-1)/$\log_{10}$N. The maximum value is (N-1)/$\log_{10}$N.
[d]Good's coverage is defined as 1-(# singleton OTU)/N.

Table S4. Reciprocal tBLASTx analysis results. Data are percentage of query contigs found hits in database. Exact contig number is listed in parenthesis.

		Database			
		10 m	100 m	1000 m	3000 m
Query	10 m	-	25.6% (4076)	20.6% (3273)	21.4% (3406)
	100 m	20.1% (5003)	-	30.1% (7479)	23.6% (5876)
	1000 m	13.8% (2967)	29.1% (6224)	-	26.3% (5641)
	3000 m	14.7% (3391)	21.3% (5000)	24.6% (5680)	-

Table S5. Pearson's correlation coefficients among oceanographic parameters. Calculation is based on the data shown in Figure S9 (Additional File 1).

	Temp	Salinity	DO	NO_3+NO_2	PO_4	SiO_2	Chl-*a*	DIC	pH
**Temp**	1	-0.881	0.918	-0.97	-0.995	-0.943	NA	-0.997	0.991
**Salinity**	-0.881	1	-0.921	0.851	0.851	0.731	NA	0.893	-0.894
**DO**	0.918	-0.921	1	-0.926	-0.913	-0.773	NA	-0.929	0.958
**NO_3+NO_2**	-0.970	0.851	-0.926	1	0.974	0.909	NA	0.973	-0.976
**PO_4**	-0.995	0.851	-0.913	0.974	1	0.958	NA	0.996	-0.989
**SiO_2**	-0.943	0.731	-0.773	0.909	0.958	1	NA	0.945	-0.906
**Chl-*a***	NA	NA	NA	NA	NA	NA	1	NA	NA
**DIC**	-0.997	0.893	-0.929	0.973	0.996	0.945	NA	1	-0.991
**pH**	0.991	-0.894	0.958	-0.976	-0.989	-0.906	NA	-0.991	1

Abbreviations: Temp, temperature; DO, dissolved oxygen; DIC, dissolved inorganic carbon; NA, not applicable.

Table S6. Top-20 globally enriched functions in ocean surfaces versus deep oceans.

Gene Family	Coefficient[a]	AIC[b]	*P*-value (BH)	Annotation
COG0404	1.76	1543.88	0	Glycine cleavage system T protein (aminomethyltransferase)
COG0697	1.09	204.54	0	Permeases of the drug/metabolite transporter (DMT) superfamily
COG0451	1.06	936.41	1.69E-321	Nucleoside-diphosphate-sugar epimerases
COG1754	2.45	644.30	1.14E-310	Uncharacterized C-terminal domain of topoisomerase IA
COG1233	2.43	663.55	1.23E-284	Phytoene dehydrogenase and related proteins
COG4664	2.27	759.76	2.13E-283	TRAP-type mannitol/chloroaromatic compound transport system, large permease component
COG0069	1.06	542.17	6.56E-254	Glutamate synthase domain 2
COG0086	1.09	1450.77	1.14E-241	DNA-directed RNA polymerase, beta' subunit/160 kD subunit
COG4663	2.30	645.89	9.50E-235	TRAP-type mannitol/chloroaromatic compound transport system, periplasmic component
COG0665	1.08	489.81	6.13E-225	Glycine/D-amino acid oxidases (deaminating)
COG1304	2.16	342.73	5.97E-219	L-lactate dehydrogenase (FMN-dependent) and related alpha-hydroxy acid dehydrogenases
COG0280	2.07	754.55	1.57E-207	Phosphotransacetylase
COG4176	2.42	488.08	1.62E-204	ABC-type proline/glycine betaine transport system, permease component
COG0623	2.44	459.71	3.35E-203	Enoyl-[acyl-carrier-protein] reductase (NADH)
COG0209	1.05	866.12	6.21E-191	Ribonucleotide reductase, alpha subunit
COG0538	2.32	492.34	5.97E-185	Isocitrate dehydrogenases
COG0411	2.05	660.97	1.98E-181	ABC-type branched-chain amino acid transport systems, ATPase component
COG0465	1.24	291.21	2.83E-172	ATP-dependent Zn proteases
COG0174	1.10	778.43	1.35E-170	Glutamine synthetase
COG1121	2.78	350.29	7.26E-170	ABC-type Mn/Zn transport systems, ATPase component

[a]The coefficient is the estimated difference between the two groups using Poisson model.
[b]The Akaike Information Criterion (AIC) represents a measure of the model fit.

Table S7. Top-20 globally enriched functions in deep oceans versus ocean surfaces.

Gene Family	Coefficient[a]	AIC[b]	*P*-value (BH)	Annotation
COG0583	-2.03	1551.69	0	Transcriptional regulator
COG0642	-1.89	3796.85	0	Signal transduction histidine kinase
COG0840	-4.09	2011.64	0	Methyl-accepting chemotaxis protein
COG0845	-1.68	1427.55	0	Membrane-fusion protein
COG1020	-3.47	759.23	0	Non-ribosomal peptide synthetase modules and related proteins
COG1309	-2.58	527.69	0	Transcriptional regulator
COG1609	-2.53	873.32	0	Transcriptional regulators
COG1629	-2.19	5948.54	0	Outer membrane receptor proteins, mostly Fe transport
COG2199	-4.57	1633.61	0	FOG: GGDEF domain
COG2200	-4.31	988.31	0	FOG: EAL domain
COG2801	-3.90	357.59	0	Transposase and inactivated derivatives
COG3436	-5.46	608.08	0	Transposase and inactivated derivatives
COG3437	-3.94	888.67	0	Response regulator containing a CheY-like receiver domain and an HD-GYP domain
COG3547	-4.92	261.00	0	Transposase and inactivated derivatives
COG3696	-4.24	327.02	0	Putative silver efflux pump
COG3706	-3.59	1531.50	0	Response regulator containing a CheY-like receiver domain and a GGDEF domain
COG4584	-4.15	109.93	0	Transposase and inactivated derivatives
COG4644	-4.82	888.31	0	Transposase and inactivated derivatives, TnpA family
COG5001	-4.38	2254.97	0	Predicted signal transduction protein containing a membrane domain, an EAL and a GGDEF domain
COG3550	-6.07	336.77	2.83E-292	Uncharacterized protein related to capsule biosynthesis enzymes

[a]The coefficient is the estimated difference between the two groups using Poisson model.
[b]The Akaike Information Criterion (AIC) represents a measure of the model fit.

Table S8. Bacterial and archaeal primer coverages. Primer coverages are estimated by using TestProb program [13] against the SILVA SSU r121 database based on SILVA Ref NR taxonomy.

Primer	Taxon (level)	# mismatch			
		0	1	2	3
Bacteria V6 forward primer	Bacteria (kingdom)	50.0%	68.2%	71.8%	82.0%
	Acidobacteria (phylum)	82.6%	89.0%	91.3%	91.7%
	Actinobacteria (phylum)	75.3%	82.1%	83.0%	83.4%
	Proteobacteria (phylum)	39.0%	75.2%	78.3%	81.2%
	Alphaproteobacteria (class)	9.1%	74.7%	77.3%	84.9%
	Betaproteobacteria (class)	6.0%	77.4%	79.2%	79.7%
	Deltaproteobacteria (class)	50.7%	86.0%	89.6%	90.9%
	Gammaproteobacteria (class)	71.0%	76.2%	76.9%	77.3%
	Cyanobacteria (phylum)	68.2%	79.2%	81.4%	83.0%
	Cyanobacteria (class)	77.5%	81.0%	81.6%	81.9%
	Bacteroidetes (phylum)	6.0%	6.7%	7.3%	81.8%
	Flavobacteriia (class)	1.8%	2.3%	2.7%	84.2%
	Firmicutes (phylum)	74.6%	80.4%	81.3%	82.0%
	Verrucomicrobia (phylum)	85.6%	89.0%	89.7%	90.3%
	Chloroflexi (phylum)	18.5%	41.5%	79.9%	90.2%
	Planctomycetes (phylum)	16.3%	50.3%	85.3%	90.9%
Bacteria V6 reverse primer	Bacteria (kingdom)	46.9%	81.4%	83.3%	83.6%
	Acidobacteria (phylum)	87.9%	92.1%	92.3%	92.3%
	Actinobacteria (phylum)	52.3%	81.9%	83.5%	83.7%
	Proteobacteria (phylum)	74.9%	81.2%	81.5%	81.6%
	Alphaproteobacteria (class)	82.5%	85.1%	85.3%	85.4%
	Betaproteobacteria (class)	71.4%	79.7%	80.1%	80.2%
	Deltaproteobacteria (class)	85.5%	91.2%	91.4%	91.5%
	Gammaproteobacteria (class)	73.3%	77.0%	77.4%	77.5%
	Cyanobacteria (phylum)	75.5%	80.2%	83.6%	84.1%
	Cyanobacteria (class)	79.7%	81.7%	82.2%	82.3%
	Bacteroidetes (phylum)	17.1%	82.6%	84.6%	84.9%
	Flavobacteriia (class)	8.0%	83.6%	85.6%	85.8%
	Firmicutes (phylum)	3.4%	78.4%	81.9%	82.2%
	Verrucomicrobia (phylum)	66.5%	89.9%	90.6%	91.0%
	Chloroflexi (phylum)	76.5%	90.1%	91.2%	91.5%
	Planctomycetes (phylum)	89.0%	81.2%	93.6%	93.7%
Archaea V6 forward primer	Archaea (kingdom)	61.7%	76.3%	77.7%	78.3%
	Euryarchaeota (phylum)	67.0%	77.2%	78.6%	79.4%
	Crenarchaeota (phylum)	57.9%	88.3%	91.7%	91.9%
	Thaumarchaeota (phylum)	52.4%	74.0%	75.1%	75.5%
Archaea V6 reverse primer	Archaea (kingdom))	70.5%	80.9%	82.7%	83.4%
	Euryarchaeota (phylum)	68.8%	80.6%	82.8%	83.7%
	Crenarchaeota (phylum)	81.5%	89.7%	90.9%	90.9%
	Thaumarchaeota (phylum)	75.5%	81.2%	81.7%	82.0%

Table S9. Bacterial 16S rRNA V6 amplicon libraries from other oceans. Sample ID is the sample name given by the VAMPS database (http://vamps.mbl.edu/).

**Sampling site**	**Depth (m)**	**Sample ID**
Azores	0	AWP_0014_2007_06_11
Azores	100	AWP_0013_2007_06_11
Azores	1200	AWP_0011_2007_06_11
Azores	3660	AWP_0009_2007_06_11
Mediterranean Sea	5	BMO_0006_2007_09_23
Mediterranean Sea	500	BMO_0011_2007_09_23
Mediterranean Sea	2000	BMO_0010_2007_09_23
HOT	10	KCK_HOT_Bv6.HOT186_10
HOT	100	KCK_HOT_Bv6.HOT186_100
HOT	1000	KCK_HOT_Bv6.HOT186_1000
HOT	3000	KCK_HOT_Bv6.HOT186_3000

References

1. Tang SL, Hong MJ, Liao MH, Jane WN, Chiang PW, Chen CB et al: Bacteria associated with an encrusting sponge (Terpios hoshinota) and the corals partially covered by the sponge. Environ Microbiol. 2011;13:1179–91.
2. Sanguinetti CJ, Dias Neto E, Simpson AJ: Rapid silver staining and recovery of PCR products separated on polyacrylamide gels. Biotechniques. 1994;17:914–21.
3. Qu T, Mitsudera H, Yamagata T: Intrusion of the North Pacific waters into the South China Sea. Journal of Geophysical Research: Oceans. 2000;105:6415–24.
4. Higginson MJ, Maxwell JR, Altabet MA: Nitrogen isotope and chlorin paleoproductivity records from the Northern South China Sea: remote vs. local forcing of millennial- and orbital-scale variability. Marine Geology. 2003;201:223–50.
5. Wong GTF, Ku TL, Mulholland M, Tseng CM, Wang DP: The SouthEast Asian time-series study (SEATS) and the biogeochemistry of the South China Sea - An overview. Deep-Sea Research Part II-Topical Studies in Oceanography. 2007;54:1434–47.
6. Emery WJ: Water types and water masses. Encyclopedia of Ocean Sciences. 2001;6:3179–87.
7. Chang YT, Hsu WL, Tai JH, Tang TY, Chang MH, Chao SY: Cold Deep Water in the South China Sea. Journal of Oceanography. 2010;66:183–90.
8. Cai WJ, Dai MH, Wang YC, Zhai WD, Huang T, Chen ST et al: The biogeochemistry of inorganic carbon and nutrients in the Pearl River estuary and the adjacent Northern South China Sea. Continental Shelf Research. 2004;24:1301–19.
9. Liu HB, Chang J, Tseng CM, Wen LS, Liu KK: Seasonal variability of picoplankton in the northern South China Sea at the SEATS station. Deep-Sea Research Part Ii-Topical Studies in Oceanography. 2007;54:1602–16.
10. Chen CTA, Wang SL, Chou WC, Sheu DD: Carbonate chemistry and projected future changes in pH and CaCO3 saturation state of the South China Sea. Marine Chemistry. 2006;101:277–305.
11. Talavera G, Castresana J: Improvement of phylogenies after removing divergent and ambiguously aligned blocks from protein sequence alignments. Systematic Biology. 2007;56:564–77.
12. Tamura K, Stecher G, Peterson D, Filipski A, Kumar S: MEGA6: Molecular Evolutionary Genetics Analysis Version 6.0. Molecular Biology and Evolution. 2013;30:2725–29.
13. Quast C, Pruesse E, Yilmaz P, Gerken J, Schweer T, Yarza P et al: The SILVA ribosomal RNA gene database project: improved data processing and web-based

tools. Nucleic Acids Research. 2013;41:D590–6.
